# A Universal Synthesis of Single‐Atom Catalysts via Operando Bond Formation Driven by Electricity

**DOI:** 10.1002/advs.202401814

**Published:** 2024-09-13

**Authors:** Xinyu Zhan, Libing Zhang, Junyoung Choi, Xinyi Tan, Song Hong, Tai‐Sing Wu, Pei Xiong, Yun‐Liang Soo, Leiduan Hao, Molly Meng‐Jung Li, Liang Xu, Alex W. Robertson, Yousung Jung, Xiaofu Sun, Zhenyu Sun

**Affiliations:** ^1^ State Key Laboratory of Organic‐Inorganic Composites College of Chemical Engineering Beijing University of Chemical Technology Beijing 100029 China; ^2^ Institute of Chemistry Chinese Academy of Sciences Beijing 100190 China; ^3^ School of Chemical and Biological Engineering Institute of Chemical Processes Institute of Engineering Research Seoul National University Seoul 08826 Republic of Korea; ^4^ School of Materials Science and Engineering Beijing Institute of Technology Beijing Key Laboratory of Environmental Science and Engineering Beijing 100081 China; ^5^ National Synchrotron Radiation Research Center Hsinchu 30076 Taiwan; ^6^ Department of Applied Physics The Hong Kong Polytechnic University Hong Kong 999077 China; ^7^ Department of Physics National Tsing Hua University Hsinchu 30013 Taiwan; ^8^ Department of Physics University of Warwick Coventry CV4 7AL UK

**Keywords:** CO_2_ reduction, electrocatalysis, electrosynthesis, nitrogen doping, single atom

## Abstract

Single‐atom catalysts (SACs), featuring highly uniform active sites, tunable coordination environments, and synergistic effects with support, have emerged as one of the most efficient catalysts for various reactions, particularly for electrochemical CO_2_ reduction (ECR). However, the scalability of SACs is restricted due to the limited choice of available support and problems that emerge when preparing SACs by thermal deposition. Here, an in situ reconstruction method for preparing SACs is developed with a variety of atomic sites, including nickel, cadmium, cobalt, and magnesium. Driven by electricity, different oxygen‐containing metal precursors, such as MOF‐74 and metal oxides, are directly atomized onto nitrogen‐doped carbon (NC) supports, yielding SACs with variable metal active sites and coordination structures. The electrochemical force facilitates the in situ generation of bonds between the metal and the supports without the need for additional complex steps. A series of MN_x_O_y_ (M denotes metal) SACs on NC have been synthesized and utilized for ECR. Among these, NiN_x_O_y_ SACs using Ni‐MOF‐74 as a metal precursor exhibit excellent ECR performance. This universal and general SAC synthesis strategy at room temperature is simpler than most reported synthesis methods to date, providing practical guidance for the design of the next generation of high‐performance SACs.

## Graphical abstract

A simple and versatile ambient condition method capable of accommodating a wide range of metals and supports for single‐atom catalyst preparation via operando bond formation driven by electricity is reported. The as‐obtained Ni single atoms using Ni‐MOF‐74 as a precursor exhibit remarkable activity for electrocatalytic CO_2_ reduction to yield CO.

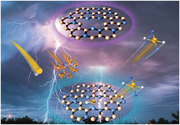

## 1 Introduction

**Scheme 1:**
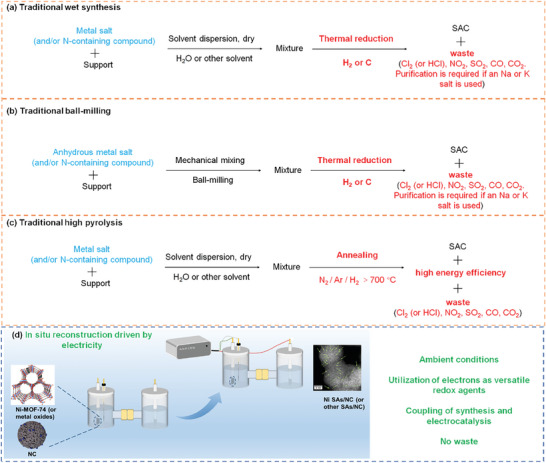
Illustration and comparison of a) traditional wet synthesis, b) ball‐milling, c) high‐temperature pyrolysis, and d) the reconstruction method reported here for preparing SACs.

Single‐atom catalysts (SACs) are formed by isolating individual metal atoms anchored on solid supports through surrounding coordination species.^[^ ^1^, ^2^ ^]^ They exhibit the highest atomic efficiency, well‐defined active centers, and specific surface areas, showcasing excellent catalytic activity and selectivity in the field of electrochemistry.^[^ ^3^, ^4^ ^]^ Strategies for synthesizing SACs include wet chemical methods, atomic layer deposition, ball milling, electrochemical deposition, and photochemical synthesis. As summarized in **Scheme** **1**, traditional methods encounter several issues, such as complex synthesis procedures (e.g., intricate single‐atom precursor preparation or defect construction) or the requirement for specialized equipment under demanding physicochemical conditions, thereby increasing the overall expense of single‐atom catalyst production.^[^ ^5^, ^6^ ^]^ Among these methods, elevated temperature treatment is the most frequently used procedure for SAC synthesis. However, this energy‐intensive process results in high preparation costs, harmful waste, and also safety risks. The obtained single‐atom structure is highly random owing to uncontrolled pyrolysis. At the same time, clusters and nanoparticles tend to form due to Ostwald ripening during the high‐temperature process. Among others, the geometry, crystallinity and phase structure of the support (especially metal oxide substrates) may be altered under high temperatures. These lead to the highly unpredictable quality of the synthesized material.^[^ ^7^ ^]^ As a result, synthesis methods that employ a low temperature and mild reaction conditions have recently attracted a wide research interest.^[^ ^8^, ^9^, ^10^ ^]^ For example, an electrodeposition method was shown to be effective for preparing SACs on metal hydroxides, metal oxides, and metal selenides.^[^ ^8^ ^]^ A click confinement strategy was adopted to synthesize SACs by utilizing cobalt porphyrin as the metal source, carbon nanotubes as the substrate, and amide bonds as the covalent bonds constructed via click reactions.^[^ ^9^ ^]^ Despite this progress, developing simple and versatile ambient condition methods capable of accommodating a wide range of metals, and supports for SAC preparation to realize high‐efficiency electrocatalytic applications is still challenging.^[^ ^11^ ^]^

During electrocatalysis, the pre‐prepared catalytic materials may undergo structural and phase changes, which are considered self‐reconstruction.^[^ ^12^ ^]^ It is an operable and universal phenomenon that occurs at room temperature, serving as a bridge that connects pristine electrode materials with the real active species involved in actual catalysis. With the continued development of in situ characterization techniques, the mechanism of the self‐reconstruction process will be further elucidated. Previous studies have focused on this aspect and have contributed to a better understanding of self‐reconstruction.^[^ ^12^, ^13^, ^14^ ^]^ Therefore, using self‐reconfiguration to prepare single atoms holds great practical significance. For instance, Zhao et al.,^[^ ^15^ ^]^ initially employed in situ leaching of Mo atoms from high‐entropy Co/Mo co‐doped NiFe LDH precursors during the OER process, thereby experimentally constructing coexistent Co dopants and cation vacancies in NiFe oxyhydroxide. This synthetic method, based on operando changes occurring in the materials during electrochemical reactions, allows the designed local structure to directly impact catalytic reactions. Additionally, Tan et al.,^[^ ^16^ ^]^ synthesized a defect‐rich copper electrode through in situ surface reconstruction under electrochemical conditions, demonstrating outstanding catalytic efficiency in the reduction of CO_2_ into alcohols. In situ spectroscopic investigations and theoretical calculations reveal that the modulation of Cu(I)/Cu(0) interfaces with abundant structural defects by in situ reconstruction would promote the formation of favorable intermediates for the subsequent coupling reaction leading to selective alcohol generation.

Herein, we have developed a novel operando electrochemical reconstruction strategy for the synthesis of SACs. Different oxygen‐containing precursors, including commercial metal oxides (such as NiO, Mn_2_O_3_, ZnO, and CdO), as well as a metal‐organic framework, M‐MOF‐74 (M═Ni, Co, Cd, and Mg), can be directly captured by the nitrogen‐doped carbon (NC obtained at 650 °C unless stated otherwise) matrix under electric drive, forming metal centers with specific coordination structures. During this process, the M─O bonds in the precursors become dangling. The low‐coordinated metal is subsequently coordinated with the N species in the NC matrix, forming in situ MN_x_O_y_ bonds in metal single atoms (designated as M SAs/NC). Note that this reconstruction method utilizes electrons as versatile redox agents and is conducted under ambient conditions, inhibiting the formation of clusters and agglomerates as well as the alteration of support geometry, crystallinity and phase structure that would occur under high temperatures. The resulting materials can be directly used as a catalytic electrode, thereby streamlining the electrode fabrication procedure. To elucidate the formation process, we provide a detailed description of the generation of Ni‐based monatomic materials through an electric drive using Ni‐MOF‐74 as the metal precursor. Combining X‐ray photoelectron spectroscopy (XPS), Raman spectroscopy, and X‐ray absorption spectroscopy (XAS) measurements, we observed the partial breaking of metal (M)─O bonds from metal precursors, which coordinated with the NC matrix to form M─N bonds. A series of synthesized M SAs/NC were used for ECR as an exemplar reaction. Due to the reconstructed axially symmetric NiN_2_O_2_ structure of Ni SACs/NC using Ni‐MOF‐74 as a metal precursor, the energy barrier for ^*^COOH formation decreased, and H_2_ evolution was inhibited to some extent. This further facilitated the electrochemical conversion of CO_2_ to CO, exhibiting extraordinary electrochemical performance and superior selectivity in ECR, demonstrating a high CO faradaic efficiency (FE_CO_) of 96.4% at −0.9 V (versus reversible hydrogen electrode, *vs*. RHE). This work provides a new approach to synthesizing SACs by taking advantage of the operando bond reconstruction between metal precursors and supports.

## 2 Results

**Figure 1:**
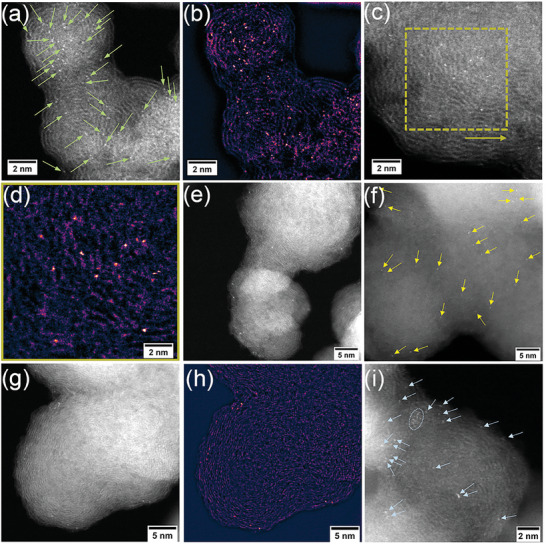
a) HAADF‐STEM image of Ni SAs/NC (using Ni‐MOF‐74 as a precursor), revealing many bright single‐atom sites of Ni decorating the carbon support (arrows). b) False color look up table of (a) following bandpass filtering, highlighting the single atom distribution. c) HAADF‐STEM image of Ni sites on Ni SAs/NC. d) False color bandpass filtered magnified view from the indicated area in (c), highlighting single atom sites. e) Bright field (BF) and high angle annular dark field (HAADF) images of Ni SAs/NC (using NiO as a precursor), the atomic number contrast HAADF‐STEM image show the bright Ni atom sites dispersed across the carbon support (indicated by arrows in (f)). g) HAADF‐STEM of Cd SAs/NC (using Cd‐MOF‐74 as a precursor), and h) false color bandpass filtered magnified view from the indicated area in (g). i) HAADF‐STEM image of Cd SAs/NC. Bright Cd atoms (indicated by arrows, cluster indicated by dotted ellipse) decorate the area.

The synthesis of M SAs/NC involves mixing an oxygen‐containing metal precursor (such as metal oxides and different types of MOF‐74) with an NC substrate, followed by a constant potential electrolysis for 1 h in a cathode chamber, resulting in in situ reconstruction, as illustrated in Scheme 1d. The details for the preparation can be found in the Supporting Information. To show the feasibility of this general synthetic approach, we take the synthesis of Ni SAs/NC using Ni‐MOF‐74 as an example for characterization and analysis. We first employed high‐angle annular dark‐field scanning transmission electron microscopy (HAADF‐STEM) (**Figure** **1**a−d) to reveal the morphology and size distribution of the catalyst. Figure 1a−d clearly shows numerous bright single dots corresponding to Ni single atoms, indicating that dispersed Ni single atoms were generated through operando electrochemical reconstruction. Energy‐dispersive X‐ray spectroscopy (EDS) elemental maps (Figure S1, Supporting Information) show Ni, N and O elements are dispersed on the surface of Ni SAs/NC. Additionally, we substituted various metal oxygen‐containing precursors for Ni‐MOF‐74 and synthesized a series of M SAs/NC (M = Ni, Co, Mg, Cd, etc.), demonstrating its versatility in obtaining a variety of SACs. Atomic dispersion of Ni in Ni SAs/NC using NiO as the metal precursor is depicted in Figure 1e,f, and atomic dispersion of Cd in Cd SAs/NC using Cd‐MOF‐74 as the metal precursor can be observed in Figure 1g−i. Corresponding EDS maps (Figures S2 and S3, Supporting Information) also verified the coexistence of metal, N, and O elements.

**Figure 2:**
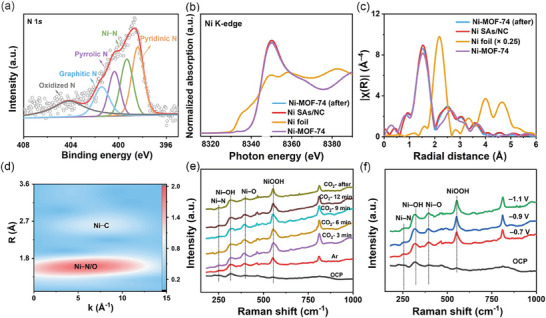
a) High‐resolution XPS spectrum of N 1*s* for Ni SAs/NC. b) Ni K‐edge X‐ray absorption near edge fine structure spectra (XANES) of Ni SAs/NC and reference samples of NiO, Ni foil, Ni‐MOF‐74, and Ni‐MOF‐74 after electrolysis (named as Ni‐MOF‐74 (after)). c) FT‐EXAFS spectra of the Ni K‐edge for Ni SAs/NC, Ni foil, Ni‐MOF‐74, and Ni‐MOF‐74 (after). d) WT‐EXAFS of Ni SAs/NC. e) In situ Raman spectra on Ni SAs/NC at −0.9 V (*vs*. RHE) with different atmospheres and electrolysis times in 0.1 M KHCO_3_ aqueous solution and f) under varied applied potentials for 10 min in CO_2_‐saturated 0.1 M KHCO_3_ aqueous solution.

A series of characterization techniques and operando technologies were employed to investigate the structure of SACs. Ni SAs/NC was selected as an example. The high‐resolution N 1*s* XPS spectrum can be deconvoluted into five types of nitrogen species (**Figure** **2**a), corresponding to oxidized N (≈403.5 eV), graphitic N (≈401.2 eV), Ni─N (≈399.5 eV), pyrrolic N (≈400.3 eV), and pyridinic N (≈398.5 eV), respectively.^[^ ^17^ ^]^ It is noteworthy that the appearance of the Ni–N peak in the high‐resolution N 1*s* spectra can be attributed to the operando formation of bonds in Ni SAs/NC.^[^ ^18^, ^19^ ^]^

Further evidence for metal–nitrogen/oxygen coordination and chemical information was obtained by XAS (Figure 2b−d and Figures S5−S10). The Ni K‐edge X‐ray absorption near‐edge structure (XANES) spectra of Ni SAs/NC compared with Ni‐MOF‐74 before and after electrocatalysis and other reference standards are shown in Figure 2b. The Ni SAs/NC shows similar XANES features with Ni‐MOF‐74 after electrolysis, indicating that Ni SAs/NC has a similar coordination structure as Ni‐MOF‐74 after electrolysis. However, the higher intensity of the white‐line feature in the XANES spectrum of Ni SAs/NC suggests a slightly higher valence state of the Ni atoms compared to Ni‐MOF‐74 after pyrolysis. Consideration of why the average oxidation state of Ni SAs/NC was partially oxidized leads us to hypothesize that partial electron transfer from the Ni centers to N sites on the NC matrix occurred due to the introduction of nitrogen,^[^ ^20^ ^]^ which aligns with the Ni 2*p* XPS results (Figure S4d, Supporting Information). The binding energy of Ni species in Ni SAs/NC is slightly shifted to a higher binding energy relative to Ni in Ni‐MOF‐74 after electrolysis, indicating that the valence of Ni in Ni SAs/NC has shifted more positively than Ni(+2) in Ni‐MOF‐74 (after) (Figure S4d, Supporting Information). Figures 2c and S5–S8 (Supporting Information) display the Fourier‐transformed extended X‐ray absorption fine structure (FT‐EXAFS) (Table S1, Supporting Information).^[^ ^21^, ^22^ ^]^ In combination with XPS (Figure 2a; Figure S4, Supporting Information) and the following in situ Raman results (Figure 2e,f; Figure S11, Supporting Information), we propose that a new structure NiN_x_O_y_ was reconstructed during electrolysis (note that it is difficult to discern N and O coordinates by EXAFS fitting due to their close atomic numbers).^[^ ^14^ ^]^ For Ni SAs/NC, no obvious Ni─Ni scattering path can be observed at ≈2.48 Å and another peak at 3.03 Å can be assigned to the second‐shell coordination, specifically the contributions of Ni─C and Ni─Ni interactions within the Ni‐MOF‐74 lattice. This confirms that the Ni atoms in the Ni SAs/NC catalysts are distributed as single atoms rather than forming clusters,^[^ ^23^ ^]^ which is further verified by the EXAFS wavelet transform (WT) of Ni SAs/NC, Ni‐MOF‐74, Ni‐MOF‐74 (after), and Ni foil (Figure 2d; Figures S9 and S10, Supporting Information). In the WT contour plot of Ni in Ni SAs/NC (Figure 2d), no intensity maximum corresponding to Ni─Ni coordination from Ni clusters can be observed, confirming the absence of Ni─Ni interactions. The single intensity maximum at ≈5.0 Å^−1^ in the WT contour plot of Ni in Ni SAs/NC (Figure 2d) can be assigned to the Ni─N/O coordination shell, which is different from Ni─Ni at 5.5 Å^−1^ (Figure S9, Supporting Information). These results suggest that the Ni atoms are anchored on the N‐doped carbon matrix by forming a metal–nitrogen coordination structure.^[^ ^24^ ^]^

To delve deeper into the dynamic evolution of Ni SAs/NC, we conducted Raman spectroscopy on NC, Ni‐MOF‐74, and Ni SAs/NC samples. Additionally, in situ Raman spectroscopy measurements of Ni SAs/NC were performed through time‐dependent electrochemical tests. The Raman spectra (Figure S11, Supporting Information) revealed that the peaks at ≈310, 400, and 550 cm^−1^ could be attributed to Ni─OH, Ni─O, and NiOOH bonds, respectively.^[^ ^19^, ^25^ ^]^ The formation of NiOOH results from the reconstruction of Ni‐MOF‐74 precursor during electrolysis, consistent with prior literature.^[^ ^26^, ^27^, ^28^ ^]^ The peaks at 1350 and 1592 cm^−1^ were assigned to the D band and G band of nitrogen‐doped carbon, respectively.^[^ ^29^ ^]^ In comparison with NC and Ni‐MOF‐74, Ni SAs/NC exhibited a prominent peak at ≈245 cm^−1^, attributed to the Ni─N bond.^[^ ^30^ ^]^ Hence, it can be concluded that the breakage of Ni─O bonds and the formation of Ni–N bonds occurred during the in situ reconstruction of Ni SAs/NC. This is also in accord with the XRD observations that the crystalline structure of Ni‐MOF‐74 was degraded with the extension of electrolysis time (Figure S12, Supporting Information).

**Figure 3:**
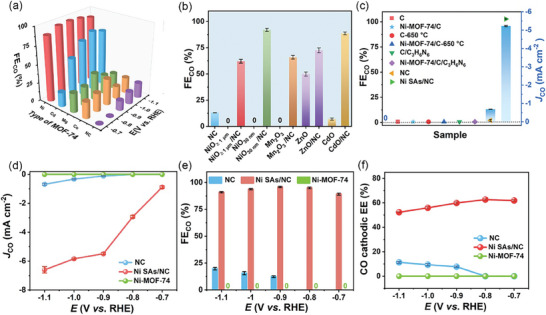
a) Faradaic efficiency toward CO formation (FE_CO_) versus applied potential on M SAs/NC catalysts derived from Ni‐, Cd‐, Mg‐, and Co‐MOF‐74. b) FE_CO_ of M SAs/NC resulting from different commercial metal oxides, including NiO with particle sizes of 30 nm and ≥ 1 µm, Mn_2_O_3_, ZnO, and CdO, as well as the pure metal oxides and NC at −0.9 V (*vs*. RHE). c) CO partial current density (*J*_CO_, bars) and FE_CO_ (symbols) of various electrode materials at −0.9 V (*vs*. RHE). d) *J*_CO_, e) FE_CO_, and f) CO cathodic EE on NC, Ni‐MOF‐74, and Ni SAs/NC at varied applied potentials. All the potential reported in this paper are versus RHE.

To validate this conclusion, we collected the in situ Raman spectra of Ni SAs/NC at −0.9 V (*vs*. RHE) over time in 0.1 M KHCO_3_ aqueous solution at room temperature under Ar and CO_2_ atmospheres (1 atm). As depicted in Figure 2e, under −0.9 V (*vs*. RHE), the Ni─N bond signal appeared in both Ar and CO_2_ atmospheres, which excludes the influence of the atmosphere on the formation of electrically driven single atoms. We also observed that the Ni─N signal persisted with increasing electrolysis time under the CO_2_ atmosphere and did not disappear after the reaction, demonstrating the stable formation of Ni─N bonds in Ni SAs/NC during the ECR process. Furthermore, we conducted in situ Raman spectroscopy measurements on Ni SAs/NC at different applied potentials (Figure 2f). The absence of the Ni─N peak at the open circuit potential (OCP), and its appearance while under applied potentials, suggest that the Ni─N bonds in Ni SAs/NC underwent in situ reconstruction during electrolysis. The formation of Ni─N in Ni SAs/NC was found to be related to the applied potential.^[^ ^30^ ^]^ The integrated Ni─N and Ni─O peak area ratio increased as a function of potential, reaching the highest value at −0.9 V (*vs*. RHE). This implies the formation of the maximum number density of Ni single atoms, which corresponds to the best ECR performance (**Figure** **3**e). Further increase of overpotential led to the decline of Ni─N and Ni─O peak ratios but an increase to the NiOOH and Ni─O peak ratios (Figure S13, Supporting Information). The decrease of the Ni─N peak fraction may result from the transformation of NiN_x_O_y_ to NiOOH at more negative potentials below −0.9 V. Based on these results, it can be speculated that the intrinsic formation mechanism of Ni SAs/NC involves the breakdown of part of the Ni─O bonds from Ni‐MOF‐74. These broken bonds then coordinate with the nitrogen atoms of NC, leading to the subsequent generation of Ni─N bonds. This intriguing mechanism of SACs formation provides a novel and feasible approach for the operando reconstruction of SACs.

By selecting various oxygen‐containing precursors, including Co, Ni, Mg, Cd‐MOF‐74, and some commercial metal oxides, we synthesized a range of catalysts and used them as catalysts for ECR. Gas chromatography (GC) and ^1^H nuclear magnetic resonance spectroscopy was utilized to analyze the ECR products, which revealed that only CO and H_2_ were detected, and no other by‐products were observed for the as‐synthesized catalysts (Figure S14, Supporting Information). The FE of CO at −0.9 V (*vs*. RHE) with various precursors was measured (Figure 3a,b). In Figure 3a, the ECR performance of catalysts with different MOF precursors was shown to be greatly enhanced compared to bare NC. Most metal oxides exhibited negligible ECR catalytic activity (Figure 3b; Figure S15, Supporting Information), while the synthesized corresponding SACs displayed significantly improved ECR performance. The development of ECR performance from negligible levels to excellence may be attributed to the reconstructed structure. These experiments further confirmed the reliability and generality of our synthesis strategy for SACs using operando electrochemical reconstruction under ambient conditions. For comparison, we attempted to fabricate NiN_x_O_y_/NC by pyrolyzing the mixture of Ni‐MOF‐74 and NC under Ar at 800 °C for 2 h. Ni‐MOF‐74 was found to be decomposed and transformed into Ni and NiO crystallites, as reflected by XRD (Figure S16a, Supporting Information). The formation of Ni and NiO clusters and agglomerates was also observed by HAADF‐STEM imaging combined with EDS analysis (Figure S16b−f, Supporting Information). Thus, the operando electrochemical reconstruction strategy reported here inhibits the formation of aggregates and any alterations of the support's geometry, crystallinity, and phase structure, which usually occur under high temperatures.

To elucidate the role of coordinated nitrogen in Ni SAs/NC, we also employed pristine carbon black (C), a mixture of C and melamine without pyrolysis (C/C_3_H_6_N_6_), and NC pyrolyzed at 600–700 °C, to mix with Ni‐MOF‐74 for preparing electrode materials. In contrast to the almost zero FE for CO formation when using the former two precursors to make Ni catalysts, the NC‐derived Ni SAs provided superior ECR performance, as shown in Figure 3c. Therefore, we speculate that the thermally derived NC matrix facilitates the formation and dispersion of Ni single atoms. The influence of pyrolysis temperature on NC and the resulting Ni SAs/NC was investigated. It was found that the Ni SAs/NC synthesized using the NC obtained at 650 °C delivered the best ECR properties (Figure S17, Supporting Information). This is ascribed to the highest Ni─N bond content in Ni SAs synthesized using the NC pyrolyzed at 650 °C as well as the largest fractions of N species in the sample. (Figure S18 and Table S2, Supporting Information) The optimal mass ratio of Ni‐MOF‐74 precursor and NC for preparing Ni SAs/NC was found to be 0.375 based on ECR activity (Figure S19, Supporting Information). The optimized Ni SAs/NC was used in subsequent characterizations and electrochemical measurements. For Ni SAs/NC, the absolute *J*_CO_ increased rapidly with the applied potential and reached 6.6 mA cm^−2^ at −1.1 V, 9.2 times higher than NC (0.7 mA cm^−2^). Additionally, as shown in Figure 3d, when the potential approached −0.8 V or a more positive potential, no CO production was observed for bare NC. This once again confirmed that Ni single atom sites were the active sites generating CO. The results showed that the FE toward CO formation against applied potential follows a volcano shape, as depicted in Figure 3e. The maximum FE_CO_ reached 96.4% on Ni SAs/NC at −0.9 V, significantly higher than that of NC (20.4% at −1.1 V). This is in stark contrast to pure Ni‐MOF‐74 which exclusively produced H_2_ at all applied potentials (Figure 3e; Figure S15, Supporting Information). Likewise, bare NC without single Ni atoms exhibited very poor CO_2_–CO conversion rates at all tested potentials (Figure 3e), further indicating that the superior CO_2_–CO selectivity of Ni SAs/NC originated from the active single Ni atoms. Furthermore, Ni SAs/NC exhibited a FE_CO_ above 90% over a broad potential window (−0.7 to −1.1 V), demonstrating the advantage of Ni SAs/NC with N and O co‐bridged metallic sites. We calculated the CO cathodic energy efficiency (EE) based on the FE for converting CO_2_ to CO and the ratio of the thermodynamic potential of the reaction to the cell voltage. The CO cathodic EE of Ni SAs/NC was as high as 63.2% at −0.8 V, markedly surpassing pure NC electrocatalysts (Figure 3f). Tafel plot tests showed that Ni SAs/NC has a markedly lower Tafel slope (≈169.7 mV dec^−1^) than bare NC (≈200.8 mV dec^−1^) (Figure S20a, Supporting Information), suggesting more rapid reaction kinetics on Ni SAs/NC. The initial electron transfer step to generate the CO_2_^−^ radical anion is likely the rate‐controlling process for CO_2_ reduction on Ni SAs/NC.^[^ ^31^ ^]^ Moreover, electrochemical impedance spectroscopy measurements (Figure S20b, Supporting Information) displayed a strikingly lower charge transfer resistance (*R*_ct_) for Ni SAs/NC compared to NC (Figure S20b, Supporting Information), thus boosting electron transfer between catalyst and CO_2_/reaction intermediates in the electrolyte.

To elucidate the impact of coordinating O and N atoms on Ni sites, density functional theory (DFT) calculations were conducted. The mechanism revealed that the ECR undergoes two proton‐electron transfer steps with two key intermediates:

(1) $$CO_{2}+H^{+}+e^{-}+*\to \;*\mathrm{COOH}$$

(2) $$*\mathrm{COOH}+H^{+}+e^{-}\to *\mathrm{CO}+H_{2}O$$

**Figure 4:**
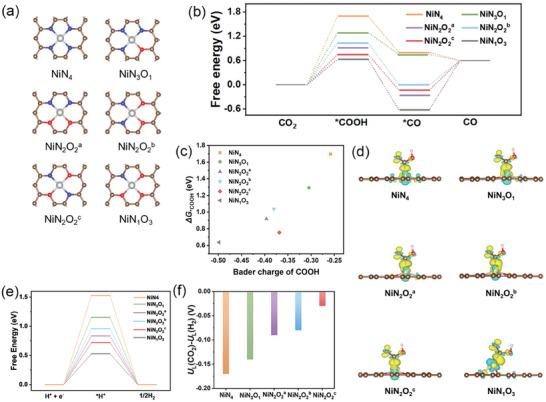
a) Different catalyst configurations of Ni sites. b) Calculated Gibbs free energy (Δ*G*) of CO_2_ reduction pathways on Ni sites with different configurations. c) The relationship between Bader charge of COOH and ΔG_*COOH_. d) The charge density difference (CDD) of *COOH and possible configurations, where cyan and yellow represent charge depletion and accumulation, respectively. e) Calculated Δ*G* of H_2_ reduction pathways on Ni sites with different configurations. f) The difference in the limiting potentials for ECR and HER (*U*_L_(CO_2_) – *U*_L_(H_2_)) on different Ni moieties.

(3) ∗CO→∗+CO

where * denotes the adsorption site on the catalyst surface.^[^ ^32^ ^]^ We considered six possible catalytic configurations as shown in **Figure** **4**a, and the corresponding Gibbs free energy of the CO_2_ pathways was calculated (Figure 4b). The adsorption of *COOH was identified as the potential‐limiting step for NiN_4_, NiN_3_O_1_, and NiN_2_O_2_. From a thermodynamic perspective, the Δ*G* for the first step (Δ*G* = *G* (*COOH) – *G* (CO_2_)) of the NiN_2_O_2_^c^ model was 0.75 eV, lower than 0.92, 1.04, 1.29, and 1.70 eV for NiN_2_O_2_^a^, NiN_2_O_2_^b^, NiN_3_O_1_, and NiN_4_, respectively. On the other hand, the rate‐determining step for NiN_1_O_3_ is a potential‐independent CO desorption with ∆*G* of 1.32 eV (Figure 4b; Table S3, Supporting Information) indicating low activity regardless of the potential. These results indicated that the NiN_2_O_2_^c^ moiety with the smallest ΔG_*COOH_ is optimal for high activity in CO production. To understand the difference in *COOH binding strengths, we calculated the d‐band centers of Ni for six catalysts (Figure S21, Supporting Information). A moderate correlation with the adsorption energies was observed (especially among those species that include a varying number of oxygen atoms), indicating that the different electronic structures of the Ni single atom affect the adsorption energies. We also calculated the Bader charge of *COOH to investigate the charge transfer between the catalysts and the adsorbed species (Figure 4c,d). Our results show that more charge was transferred to *COOH as ΔG_*COOH_ decreased, suggesting that CO_2_ can be better activated to *COOH.^[^ ^29^ ^]^

We compared these moieties for the hydrogen evolution reaction (HER) pathway on Ni sites with different configurations, as shown in Figure 4e. According to the reported literature,^[^ ^33^ ^]^ the catalytic selectivity can be probed by the difference in thermodynamic limiting potentials for ECR and HER, which can be calculated as follows; ∆*U* = *U*_L_(CO_2_) – *U*_L_(H_2_), where *U*_L_ represents the minimum potential at which all reaction steps exhibit a downhill free energy. Figure 4f shows the ∆*U*s of 5 catalysts, except for the NiN_1_O_3_ which has a potential‐independent rate‐determining step. Since a more positive ∆*U* means higher selectivity for CO_2_ reduction, we conclude that NiN_2_O_2_^c^ exhibits the best selectivity for ECR compared to any other moieties. To summarize these calculations, we have proposed the processes of site formation and catalytic reactions under electroreduction conditions, which involve the in situ reconstruction of single‐atom catalysts on NC substrates; the initial part of the Ni─O structure first breaks and reconstructs with the N in the NC matrix, further participating in the ECR relying on the generated N_2_─Ni─O_2_ structure. Subsequently, CO_2_ activation is facilitated by the binding of CO_2_ molecules and Ni atoms, followed by the formation of *COOH with the help of H^+^/*e* ^−^ charge transfer. Afterward, through the second proton‐coupled transfer *COOH undergoes reduction to *CO and desorbs from the catalytic site.

## 3 Conclusion

We have developed a novel approach for the operando synthesis of SACs by electrochemical reconstruction of the metal‐containing precursor, facilitating its integration into the nitrogen‐doped carbon support. We demonstrate this room temperature synthesis approach is generally applicable, with a range of metal M precursors (M═Ni, Co, Mg, Cd, etc.) able to form single‐atom active sites through coordination between N and O, with the N atoms from the NC matrix and the O from the oxygen‐containing precursors, forming MN_x_O_y_ active sites in M SAs/NC. Our general approach is versatile, allowing for flexibly customizing single‐atom catalysts for different coordination environments and their design for specific reactions. Notably, using Ni‐MOF‐74 as a precursor, the synthesized Ni SACs exhibit excellent ECR activity, comparable to other single‐atom ECR electrocatalysts reported in the literature. This work presents an innovative atomic‐level engineering pathway for preparing new atomically dispersed metal electrocatalysts through an in situ electrochemical reconstruction method.

## Conflict of Interest

The authors declare no conflict of interest.

## Supporting information

Supporting Information

## Data Availability

The data that support the findings of this study are available from the corresponding author upon reasonable request.
