# Supplementary material for: A Universal Synthesis of Single‐Atom Catalysts via Operando Bond Formation Driven by Electricity
Source: Adv Sci (Weinh). 2024 Sep 13;11(41):2401814. doi: 10.1002/advs.202401814 (PMC11835128; doi:10.1002/advs.202401814)
Supplement: Supplementary file 1 — Supporting Information [file ADVS-11-2401814-s001.docx]

Supporting Information

A Universal Synthesis of Single-atom Catalysts via Operando Bond Formation Driven by Electricity

Xinyu Zhan,^[a],†^ Libing Zhang,^[b],†^ Junyoung Choi,^[c]^ Xinyi Tan,^*[d]^ Song Hong,^[a]^ Tai-Sing Wu,^[e]^ Pei Xiong,^[f]^ Yun-Liang Soo,^[g]^ Leiduan Hao,^[a]^ Molly Meng-Jung Li,^[f]^ Liang Xu,^[a]^ Alex W. Robertson,^[h]^ Yousung Jung,^*[c]^ Xiaofu Sun,^*[b]^ and Zhenyu Sun^*[a]^

^[a]^ X. Zhan, Dr. S. Hong, Dr. L. Hao, Dr. L. Xu, and Prof. Dr. Z. Sun

State Key Laboratory of Organic-Inorganic Composites, College of Chemical Engineering, Beijing University of Chemical Technology, Beijing 100029, China

^[b]^ L. Zhang, Prof. Dr. X. Sun

Institute of Chemistry, Chinese Academy of Sciences, Beijing 100190, China

^[c]^ J. Choi, and Prof. Dr. Y. Jung

School of Chemical and Biological Engineering, Seoul National University, Seoul 08826, Republic of Korea

^[d]^ Prof. Dr. X. Y. Tan

School of Materials Science and Engineering, Beijing Institute of Technology, Beijing Key Laboratory of Environmental Science and Engineering, Beijing 100081, China

^[e]^ Dr. T. -S. Wu

National Synchrotron Radiation Research Center, Hsinchu 30076, Taiwan

^[f]^ P. Xiong and Dr. M. M. -J. Li

Department of Applied Physics, The Hong Kong Polytechnic University, Hong Kong, China

^[g]^ Prof. Dr. Y. -L. Soo

Department of Physics, National Tsing Hua University, Hsinchu 30013, Taiwan

^[h]^ Dr. A. W. Robertson

Department of Physics, University of Warwick, Coventry CV4 7AL, UK

*Corresponding Authors.

Email: sunzy@mail.buct.edu.cn; sunxiaofu@iccas.ac.cn; yousung.jung@snu.ac.kr; xinyitan@bit.edu.cn

^†^ These authors contribute equally to this work.

Experimental Section

Chemicals and reagents

Nickel(II) nitrate hexahydrate (Ni(NO_3_)_2_·6H_2_O), potassium hydroxide (KOH), and cadmium(II) oxide (CdO) were purchased from Macklin. Cobalt(II) nitrate hexahydrate (Co(NO_3_)_2_·6H_2_O), 2,5-dihydroxyterephthalic acid, N,N-dimethylformamide (DMF), nickel oxide, and nano zinc oxide (ZnO) were obtained from Aladdin. Cadmium(II) nitrate tetrahydrate (Cd(NO_3_)_2_·4H_2_O) was purchased from damas-beta. Magnesium(II) nitrate hexahydrate (Mg(NO_3_)_2_·6H_2_O) and potassium bicarbonate (KHCO_3_) were obtained from Innochem. Manganese(Ⅲ) oxide (Mn_2_O_3_) was purchased from bide pharmatech Ltd. Nano nickel(II) oxide (NiO) was bought from Meryer. Methanol, Nafion solution (5 wt%), and melamine were purchased from Sigma-Aldrich. Ethanol was provided by Beijing Chemical Works. Carbon black (CABOT VXC-72R). Nafion membranes were provided by Alfa Aesar. Ultrapure water (18.2 MΩ•cm) was obtained from a Millipore system for preparing the sample solutions, electrolytes, and for washing. High purity nitrogen gas (99.9999%) and carbon dioxide (99.999%) were purchased from Beijing Haipu Gas Co., Ltd. All chemicals were analytical grade and used without further purification.

Metal precursors employed and their synthesis

Commercial metallic oxides NiO, CdO, Mn_2_O_3_, and ZnO were directly used as precursors without any modification for preparation of Ni, Cd, Mn, and Zn SAs/NC respectively. Alternatively, Ni-MOF-74, Cd-MOF-74, Co-MOF-74, and Mg-MOF-74 were also applied for preparation of Ni, Cd, Co, and Mg SAs/NC respectively. The synthesis of M-MOF-74 was conducted following previous reports with some modifications.^[1]^ Taking the synthesis of Ni-MOF-74 as an example, nickel(II) nitrate hexahydrate and 2,5-dihydroxyterephthalic acid with a mole ratio of 3:1 were dissolved in a 10 mL mixed solution (*V*_DMF_:*V*_Methanol_:*V*_H2O_=15:1:1) and stirred at room temperature for 10 minutes. Then, the mixture was transferred into a Teflon-lined stainless-steel autoclave and heated at 100 °C for 24 hours. Finally, Ni-MOF-74 was collected by centrifugation, washed with DMF and methanol several times, dipped in methanol for one day, and then dried at 120 °C overnight under vacuum. The synthesis of Co-MOF-74, Mg-MOF-74, and Cd-MOF-74 followed the same procedure as Ni-MOF-74, except that nickel(II) nitrate hexahydrate was replaced with magnesium(II) nitrate hexahydrate, cobalt(II) nitrate hexahydrate, and cadmium(II) nitrate tetrahydrate, respectively.

## Synthesis of NC

For the synthesis of NC, 120 mg of carbon black and 300 mg of melamine were dispersed in 5 mL of ethanol and stirred at room temperature for 2 hours. Subsequently, the mixture was dried at 60 °C under vacuum. Finally, the prepared sample was annealed at a temperature (e.g., 600, 650, and 700 °C) under a nitrogen atmosphere for 2 hours with a ramp rate of 5 °C min^−1^. After cooling down to room temperature, NC powder was obtained.

## Synthesis of M SAs/NC

0.45 mg of a metal precursor and 1.2 mg of NC were dispersed in 90 μL and 240 μL of a mixture of isopropanol, deionized water, and Nafion solution (5 wt%) with a corresponding volume ratio of 120: 120: 1.2, respectively, under bath untrasonication for 30 minutes to form homogeneous suspensions. The resulting suspensions were mixed and then deposited onto a Toray carbon paper working electrode with an area of 1.2 cm × 1.0 cm, followed by drying at 60 °C. Subsequently, electrochemical treatment was carried out in a 0.1 M CO_2_-saturated KHCO_3_ solution using an H-type cell with two compartments separated by a Nafion 117 membrane under a specific potential (from ‒0.7 to ‒1.1 V *vs*. RHE) to in situ reconstruct M SAs/NC. The anolyte used here was 0.1 M H_2_SO_4_. Toray carbon fiber paper with a size of 1.2 cm × 1.0 cm was used as the working electrode. Pt wire and Ag/AgCl electrodes were used as the counter electrode and reference electrode, respectively. The potentials were controlled by an electrochemical working station (CHI 760E, Shanghai CH Instruments Co., China). All potentials stated in this work are relative to the reversible hydrogen electrode (RHE) scale unless otherwise mentioned. All potentials in this study (without compensation of voltage drop due to solution resistance, iR) were measured against the Ag/AgCl reference electrode (in saturated KCl solution) and converted to the RHE reference scale by:

*E* (*vs*.⋅RHE) = *E* (*vs*. Ag/AgCl) + 0.197 + 0.0591 × pH

The fraction of NiN_x_O_y_ in M SAs/NC can be tuned by changing the applied potentials and electrolysis time. By altering the type and amount of the metal precursor while keeping the amount of NC constant, different M SAs/NC samples were obtained.

Characterization

Powder X-ray diffraction (XRD) patterns were obtained using a D/MAX-RC diffractometer operated at 30 kV and 100 mA with Cu Kα radiation (*λ* = 0.15418 nm) at a scanning rate of 5° min^−1^. X-ray photoelectron spectroscopy (XPS) experiments were conducted using a Thermo Scientific ESCALAB 250Xi instrument. The instrument was equipped with an S5 electron flood and a scanning ion gun. The binding energy was corrected for surface charging by referencing the C 1*s* peak of contaminant carbon at 284.8 eV. Spectral fitting was performed using the XPS Peak41 program with Gaussian functions after subtracting a Shirley background. For post-mortem XPS analysis of samples after electrolysis, Ni-MOF-74 and Ni SAs/NC were primarily used as cathode materials for CO_2_ electrolysis. After electrolysis, the electrode materials were collected, washed repeatedly with ethanol, and dried at 60 °C under vacuum. The dried powder was carefully sealed for XPS measurements.

Scanning electron microscopy (SEM) was performed using an S-4800 microscope with a 3 kV accelerating voltage. Transmission electron microscopy (TEM) and aberration-corrected high-angle annular dark-field scanning TEM (HAADF-STEM) were conducted on a JEOL ARM200 microscope with a 200 kV accelerating voltage. STEM samples were prepared by depositing a droplet of suspension onto an Au grid coated with a lacey carbon film. In situ Raman spectroscopy (Horiba Labram HR Evolution Raman System) was carried out using a 532-nm excitation laser, and the signals were recorded with a 20 s integration time by averaging two scans. All Raman measurements were performed using a modified flow cell with the electrochemical workstation (CHI 660E) under real CO_2_ reduction electrolysis conditions

The Ni K-edge X-ray absorption near-edge fine structure (XANES) and extended X-ray absorption fine structure (EXAFS) spectra were measured at TLS 07A of Taiwan Light Source, NSRRC. The energy resolution (Δ*E*/*E*) for the incident X-ray photons was approximated to be 1.4 × 10^−4^ eV by using an Si(111) double crystal monochromator. The Ni measurements were carried out in the fluorescence mode.^[2]^ The Ni SAs/NC sample used for XAS measurements resulted from a metal precursor of Ni-MOF-74 and NC with a mass ratio of 3:8. Ni SAs/NC loaded on a carbon paper working electrode was generated after 1 hour of electrolysis at −0.9 V (*vs*. RHE). The Ni SAs/NC-loaded electrode was disconnected from the cell and immersed into ethanol in a beaker and subjected to mild bath ultrasonication for 2 min. The detached precipitate was collected by ultracentrifugation and dried at 60 °C. The resulting powder was used for XAS measurements. The Ni-MOF-74 (after) sample used for XAS measurements was prepared by using similar conditions as Ni SACs/NC (using Ni-MOF-74 as a precursor) except the absence of NC.

Electrochemical measurements

Electrochemical measurements were conducted at room temperature and atmospheric pressure using a CHI 760E electrochemical analyzer (CH Instruments, Inc., Shanghai). An H-type cell, separated by a cation exchange membrane (Nafion^®^117, Alfa Aesar), was employed for CO_2_ electrochemical reduction. The saturated Ag/AgCl electrode and platinum mesh served as the reference and counter electrodes, respectively. All potentials in this study were measured against the Ag/AgCl reference electrode (in saturated KCl solutions) and converted to the RHE reference scale. The pH value for 0.1 M KHCO_3_ saturated with pure CO_2_ is 6.8 according to the literature.^[3]^ CO_2_ with a flow rate of 10 mL⋅min^−1^ was purged into the KHCO_3_ solution for at least 30 min to remove residual air in the reservoir, then controlled potential electrolysis was performed at each potential for 60 min.

For H-type cell tests, M SAs/NC was directly used for CO_2_ electrochemical reduction. For other catalysts, such as NC and Ni-MOF-74, 1.2 mg of NC or Ni-MOF-74 was dispersed in 240 μL of a mixture of isopropanol, deionized water, and Nafion solution (5 wt%) with a corresponding volume ratio of 120:120:1.2. The mixture was bath-ultrasonicated for 30 minutes to form a homogeneous suspension. Subsequently, the suspension was loaded onto a Toray carbon paper working electrode with an area of 1.2 cm × 1.0 cm and dried at 60 °C. The resulting Ni sample was named as Ni-MOF-74 (after).

Gas-phase products were detected using gas chromatography (GC) with a thermal conductivity detector (TCD, for detecting H_2_) and a flame ionization detector (FID, for detecting CO). Gas-phase components were detected after 1 hour. Liquid products were analyzed by ^1^H NMR (nuclear magnetic resonance) using dimethyl sulfoxide (DMSO) as an internal standard. The water peak was suppressed using a solvent pre-saturation technique.^[4]^

The faradaic efficiencies of products were calculated as follows:

$$\text{FE=}\frac{\text{Z}\text{ }\text{×}\text{ }\text{n}\text{ }\text{×}\text{ }\text{F}}{\text{Q}_{\text{total}}}$$

FE: faradaic efficiency for CO or H_2_;

*Z*: number of electrons transferred (*Z* = 2 for CO and H_2_ production);

*n*: number of moles for a given product;

*F*: Faraday's constant (96485 C mol^−1^);

*Q*_total_: all the charge passed throughout the electrolysis process, which can be measured by calculating the curve area of current density *vs*. time plot.

Partial current density of CO (*J*_CO_) was calculated according to the following formula:

$$\text{J}_{\text{CO}}\text{ }\text{=}\text{ }\text{J}\text{ }\text{×}\text{ }\text{FE}_{\text{CO}}$$

*J*_CO_: partial current density for CO production;

*J*: total current density;

FE_CO_: faradaic efficiency for CO.

Cathodic energy efficiency (EE_ca._) was calculated as below:

$$\text{EE}_{\text{ca. }}\text{=}\text{E}_{\text{eq,cell}}\text{ }\text{×}\text{ }\text{FE/(}\text{E}_{\text{eq,cell}}\text{ }\text{+}{\text{ }\text{η}}_{\text{cathode}}\text{)}$$

*E*_eq, cell_ is the thermodynamic equilibrium potential between the anode and cathode reactions, i.e., $\text{E}_{\text{eq, cell}}\text{ }\text{=}{\text{ }\text{E}}_{\text{eq, anode}}\text{ }\text{+}\text{ }\text{(-}\text{E}_{\text{eq, cathode}}\text{)}$.

In this paper, anodic reaction is oxygen evolution reaction, where *E*_eq, anode_ = 1.23 V (*vs*. RHE). And cathodic reaction is the conversion CO_2_ to CO, in which *E*_eq, cathode_= −0.11 V (*vs*. RHE). *η*_cathode_ is the cathode overpotential.

Computational methods

We performed spin-polarized DFT calculations using the Vienna ab initio simulation package (VASP).^[5]^ The Perdew-Burke-Ernzerhof (PBE) functional was adopted within the generalized gradient approximation.^[6]^ The projector augmented wave (PAW) method was employed with a plane-wave basis cutoff energy of 500 eV.^[7]^ To describe weak van der Waals interactions, Grimme's D3 method with zero-damping was used.^[8]^ A 5×5 hexagonal supercell of monolayer graphene doped with nitrogens was used to model Ni SAs/NC, along with a vacuum layer of 20 Å to prevent interactions between periodic images. The Brillouin zone was sampled in the Monkhorst-Pack scheme with a 3×3×1 grid.^[9]^ The convergence criteria for electronic self-consistent calculations and relaxations were set to 10^−5^ eV and 0.05 eV/Å, respectively. The free energy of each reaction intermediate was obtained as follows:

*G* = EDFT + EZPE – *TS*

where EDFT, EZPE, *T*, and *S* are the total energy calculated by DFT calculations, zero-point energy, temperature (298.15 K), and entropy, respectively. EZPE was obtained by using Vaspkit 1.3.3.^[10]^


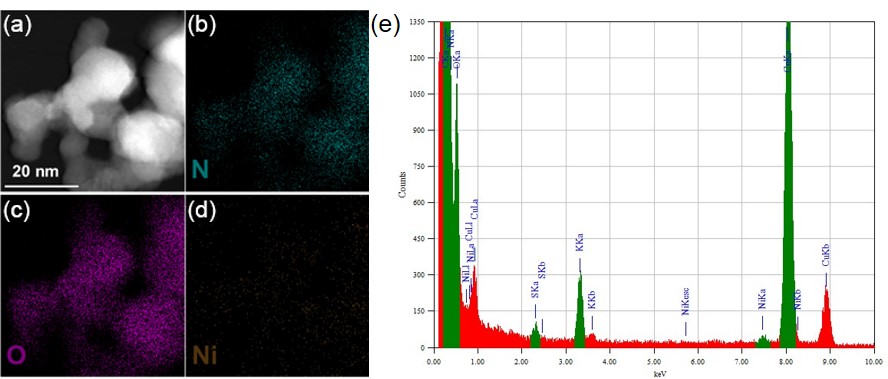


**Figure S1.** (a) HAADF-STEM image of Ni SAs/NC (using Ni-MOF-74 as a precursor) and corresponding EDS elemental maps of N (b), O (c), and Ni (d). (e) EDS spectrum of the region shown in image (a).


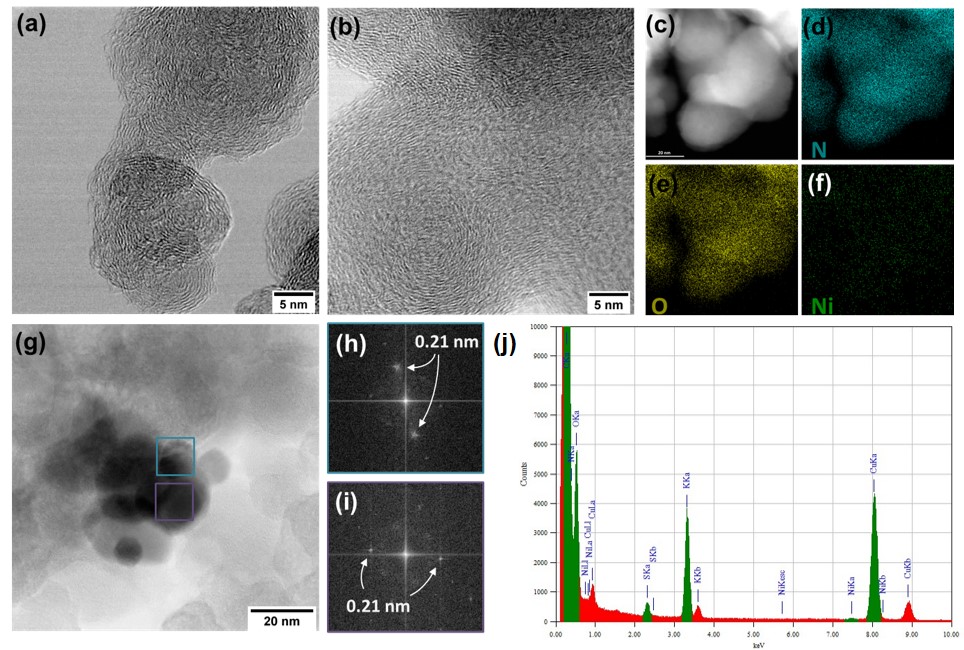


**Figure S2.** (a, b) Bright field (BF) images of Ni SAs/NC (using NiO as a precursor). (c) HAADF-STEM image of Ni SAs/NC (using NiO as a precursor) and corresponding EDS elemental maps of N (d), O (e), and Ni (f). (g) BF-STEM image of a cluster of NiO nanoparticles on the carbon support, with (h, i) fast Fourier transforms taken from the colored boxed areas. Reflections from the FFTs correspond to the (200) planes for NiO. (j) EDS spectrum of the region shown in image (g).


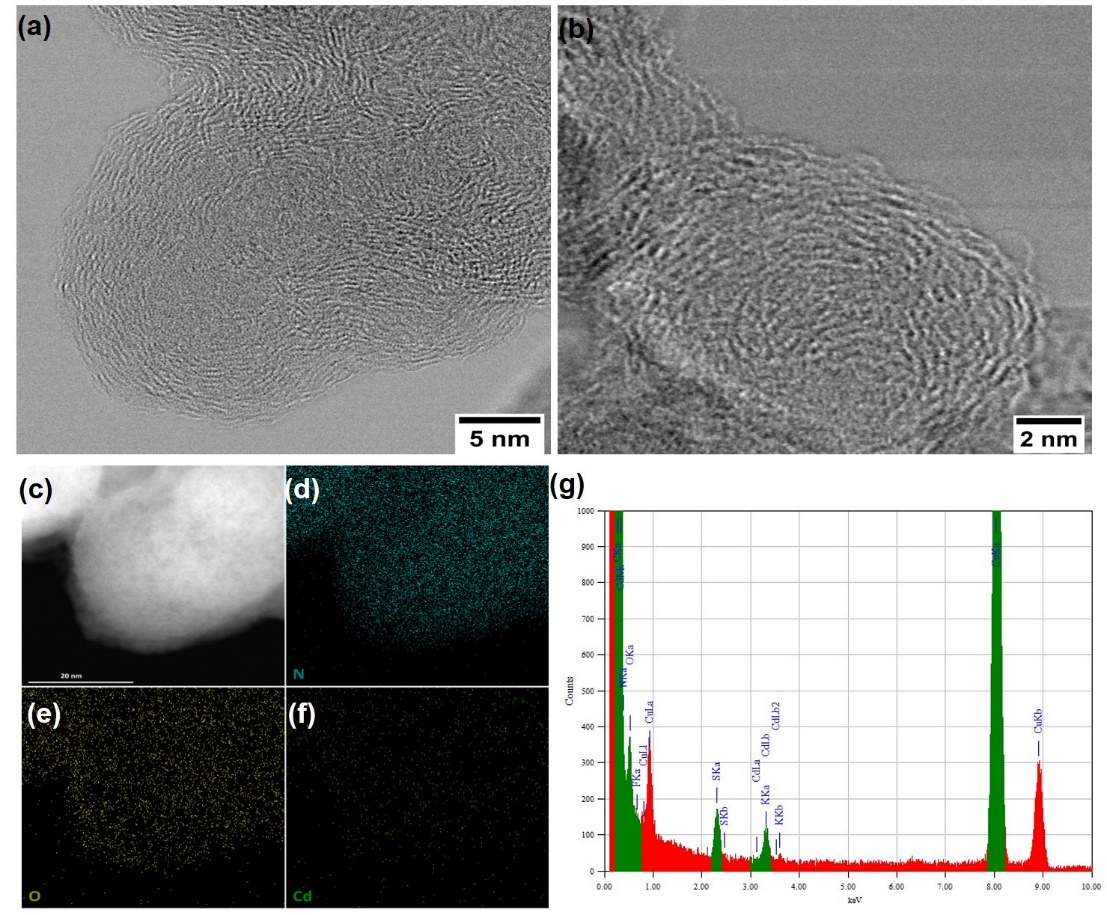


**Figure S3.** (a, b) Bright field (BF) of Cd SAs/NC (using Cd-MOF-74 as a precursor). (c) High angle annular dark field (HAADF) images of Cd SAs/NC (using Cd-MOF-74 as a precursor) and corresponding EDS elemental maps of N (d), O (e), and Cd (f). (g) EDS spectrum of the region shown in image (a).


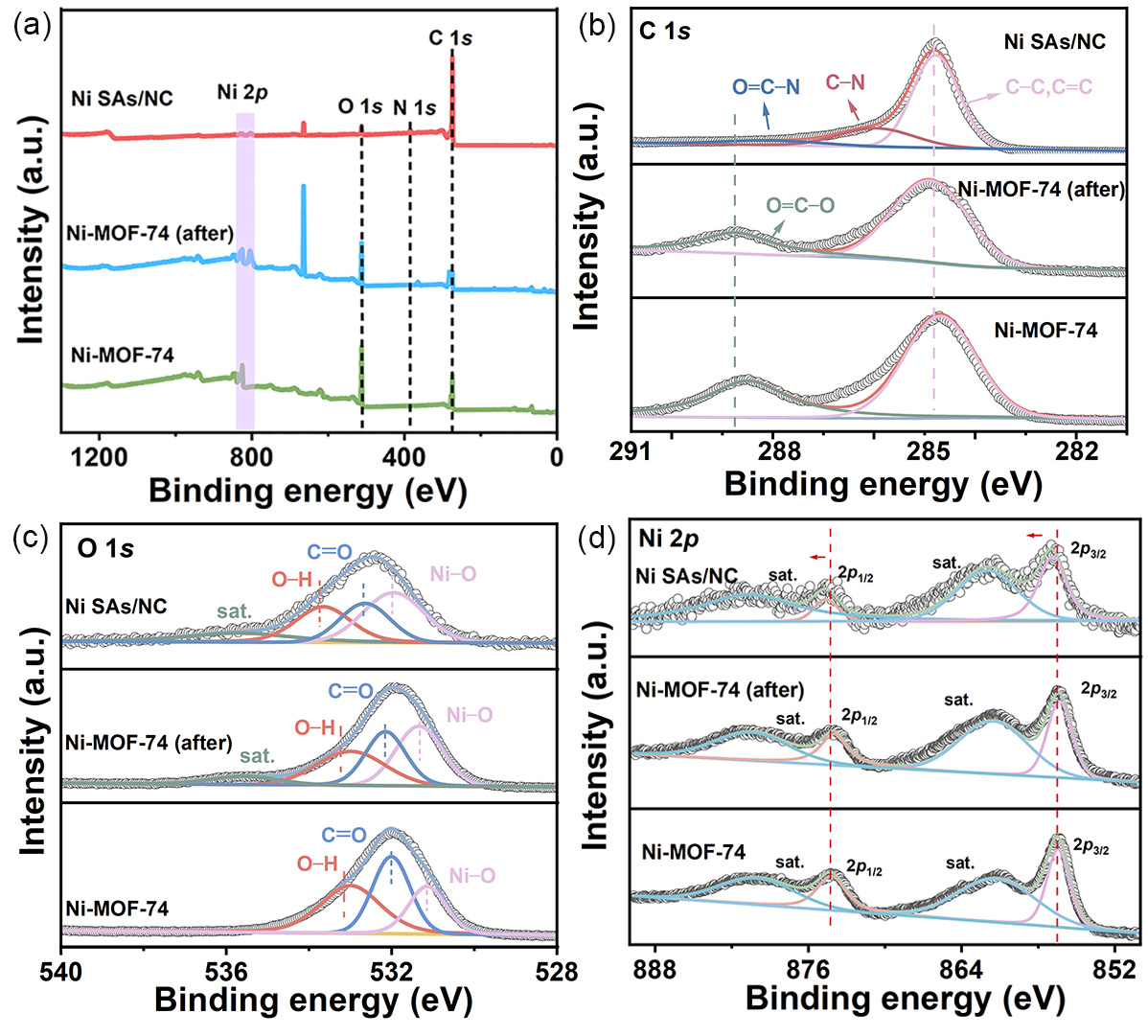


**Figure S4.** (a) Wide-survey, (b) C 1*s*, (c) O 1*s*, and (d) Ni 2*p* XPS spectra of Ni-MOF-74 before and after electrolysis along with Ni SAs/NC.


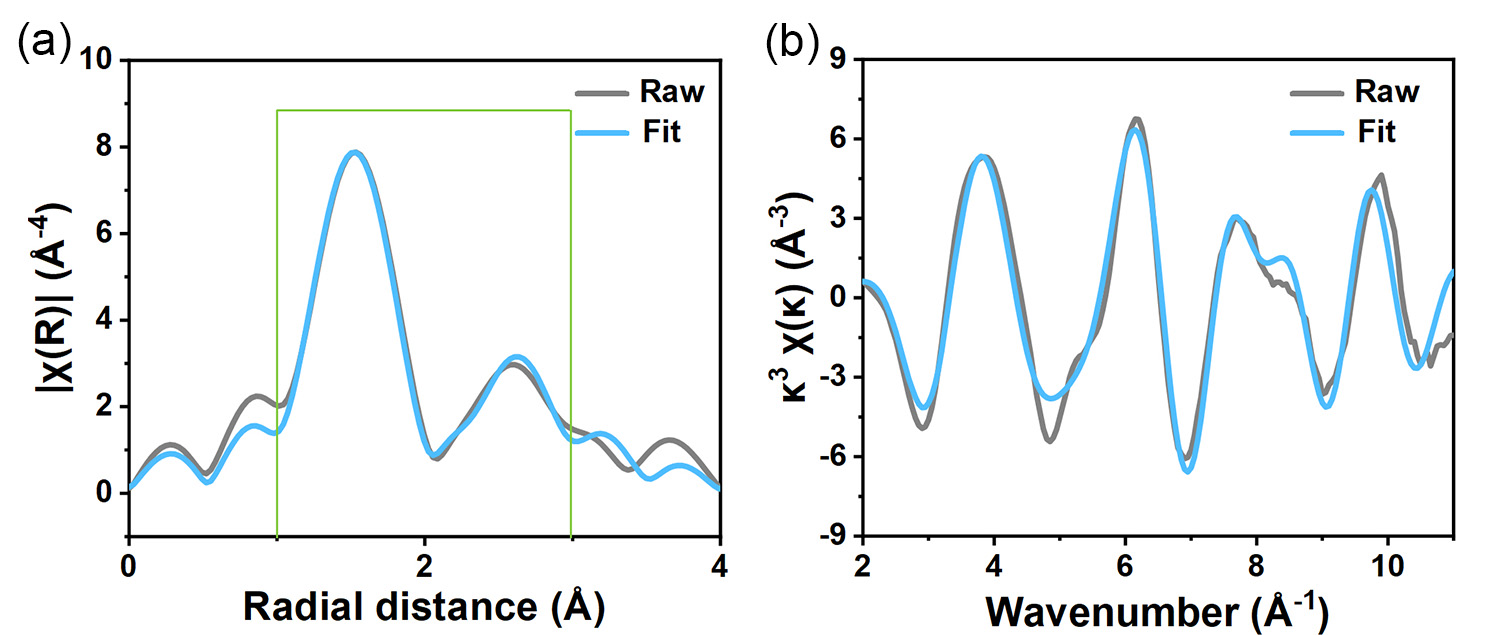


**Figure S5.** (a) FT-EXAFS spectra of the Ni K-edge for Ni-MOF-74 (after). (b) Ni K-edge EXAFS oscillations in *k* space (k^3^-weighting) of Ni-MOF-74 (after).


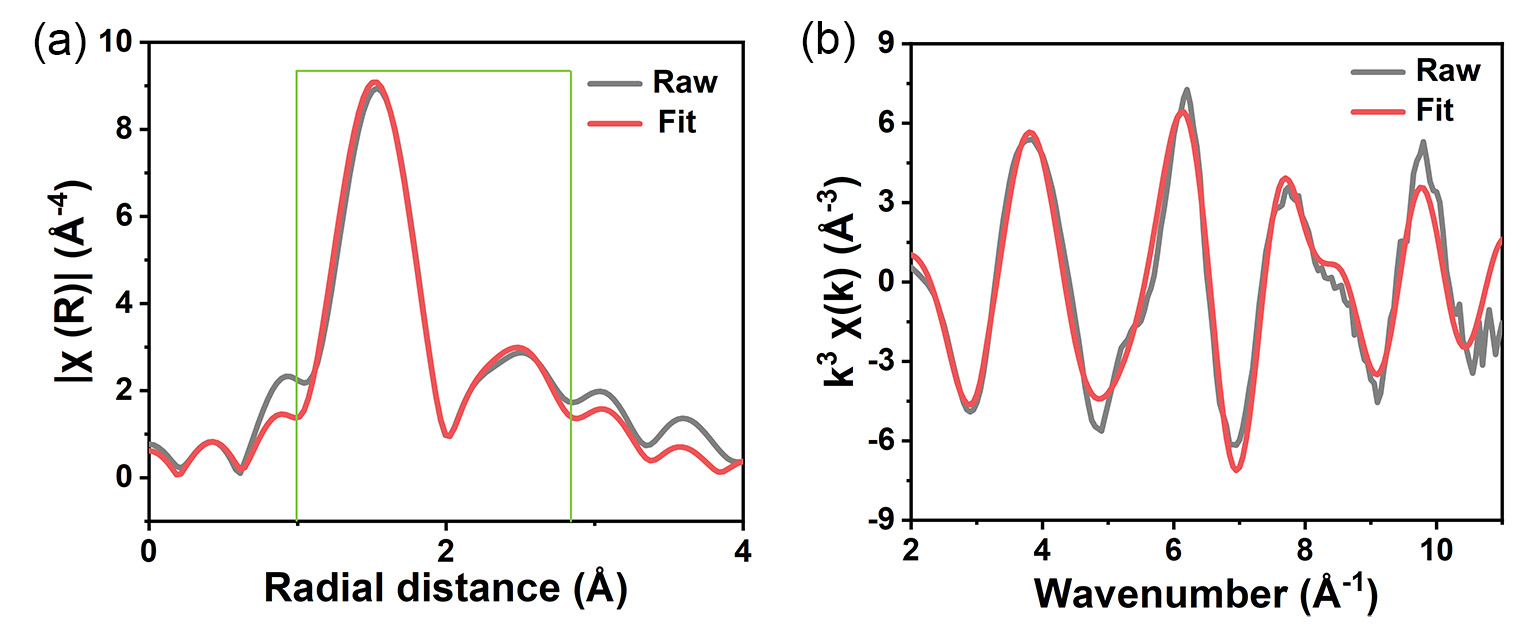


**Figure S6.** (a) FT-EXAFS spectrum of the Ni K-edge for Ni SAs/NC. (b) Ni K-edge EXAFS oscillations in *k* space (k^3^-weighting) of Ni SAs/NC.


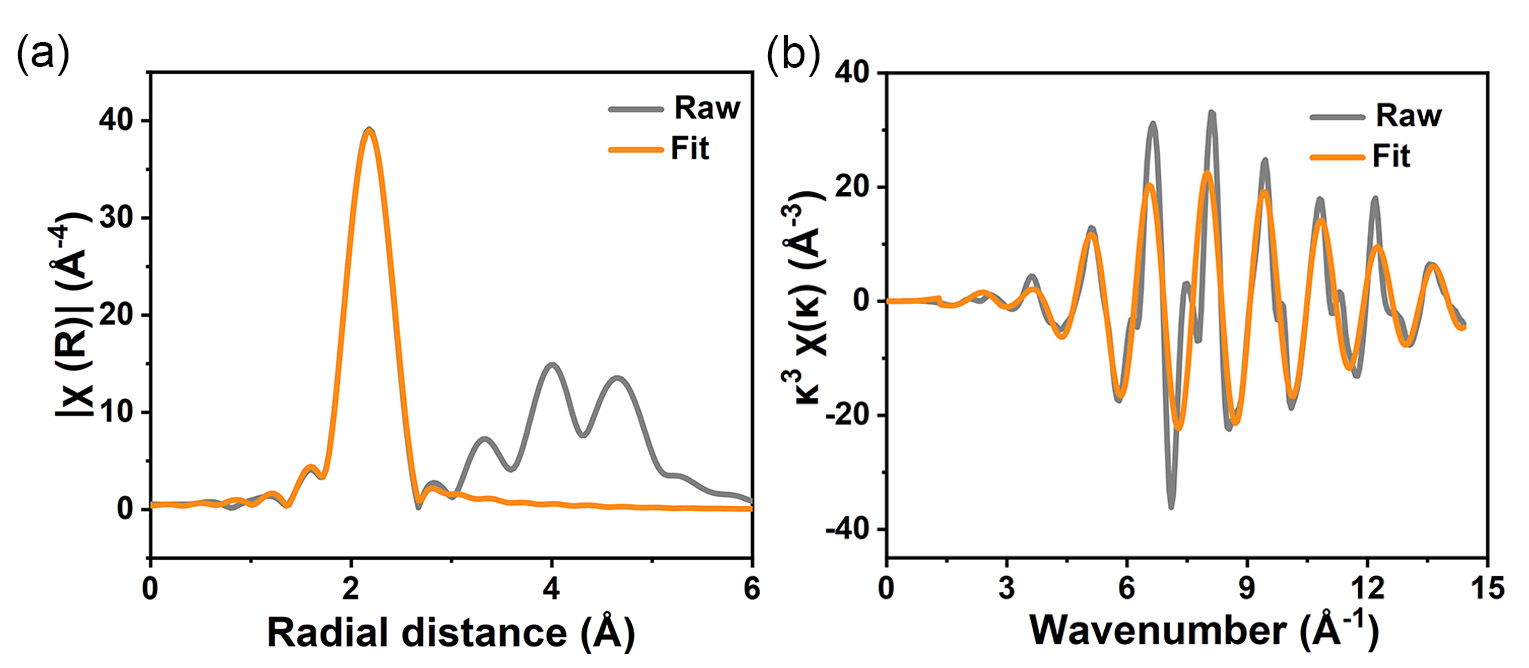


**Figure S7.** (a) FT-EXAFS spectrum of the Ni K-edge for Ni foil. (b) Ni K-edge EXAFS oscillations in *k* space (k^3^-weighting) of Ni foil.

**
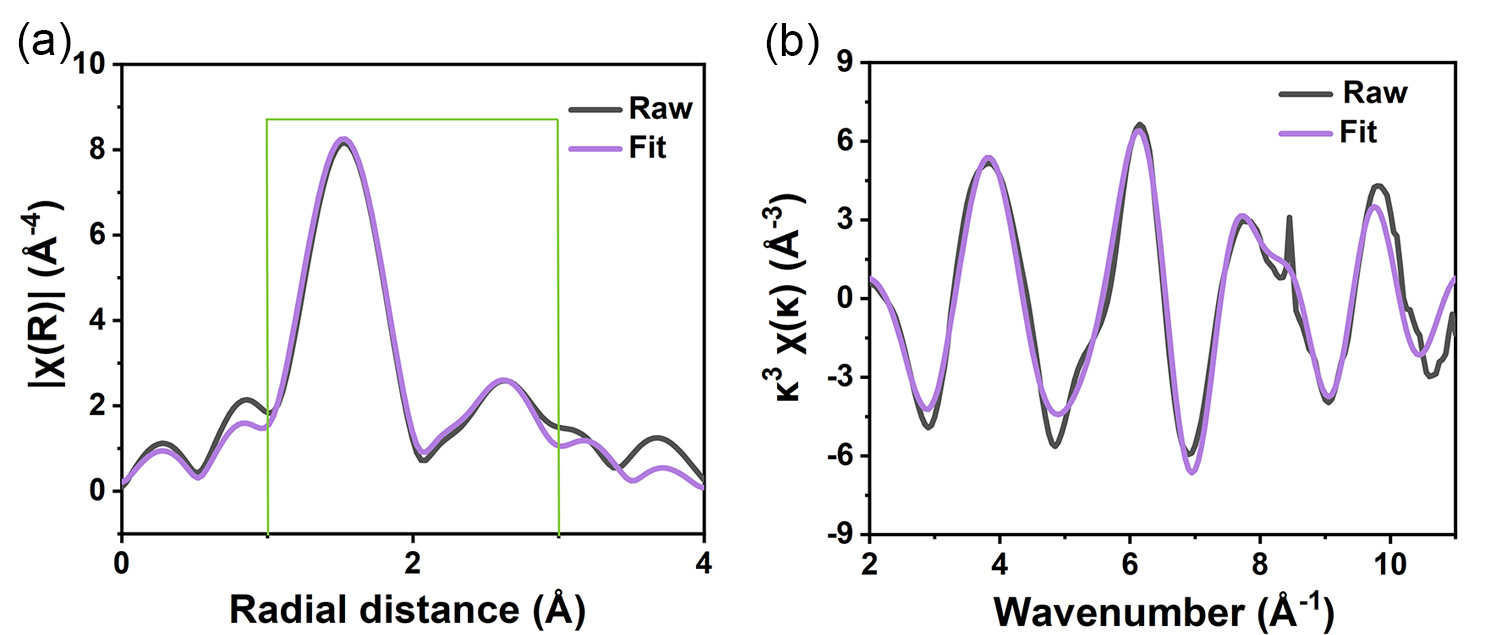
**

**Figure S8.** (a) FT-EXAFS spectrum of the Ni K-edge for Ni-MOF-74. (b) Ni K-edge EXAFS oscillations in *k* space (k^3^-weighting) of Ni-MOF-74.


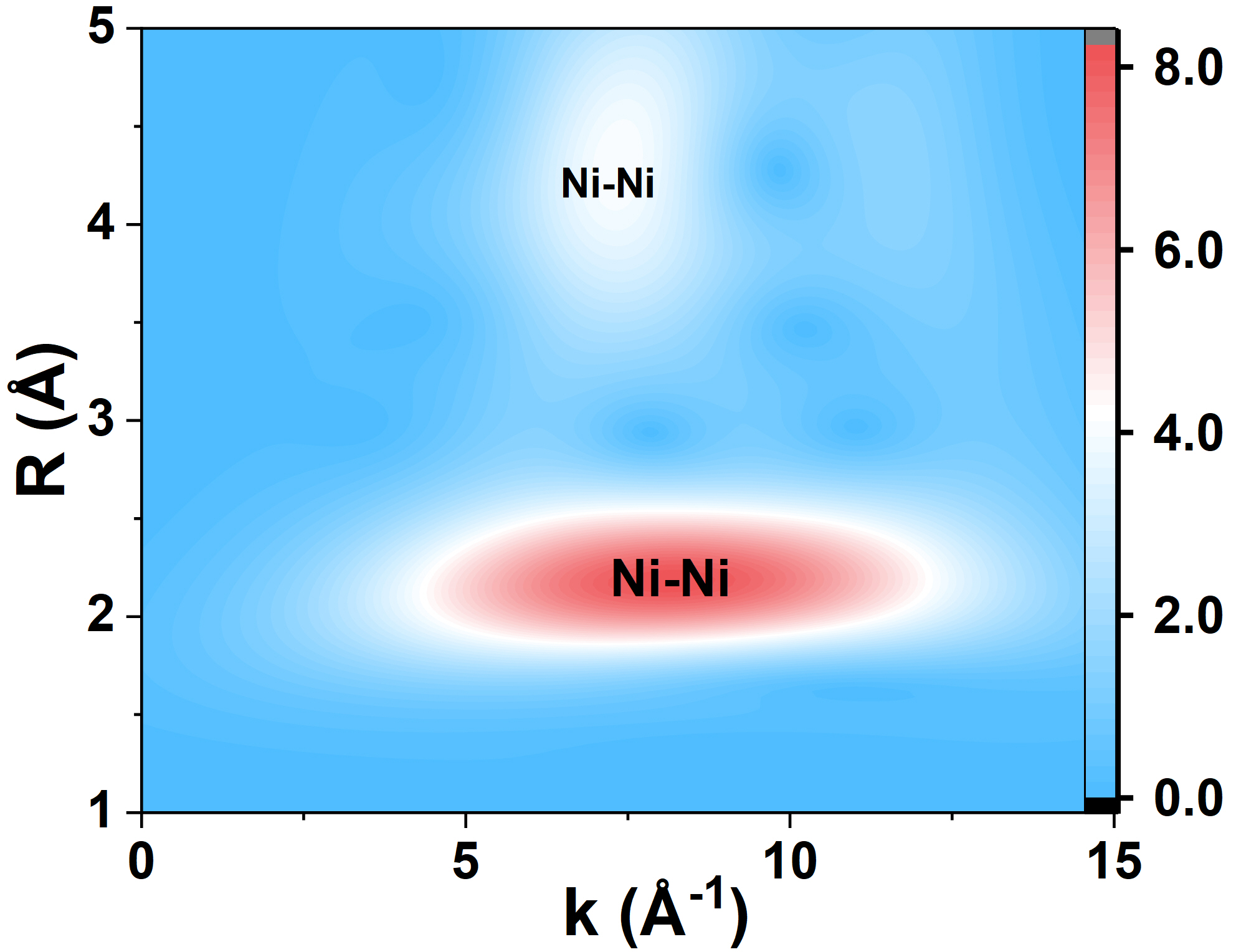


**Figure S9.** WT-EXAFS of Ni foil.


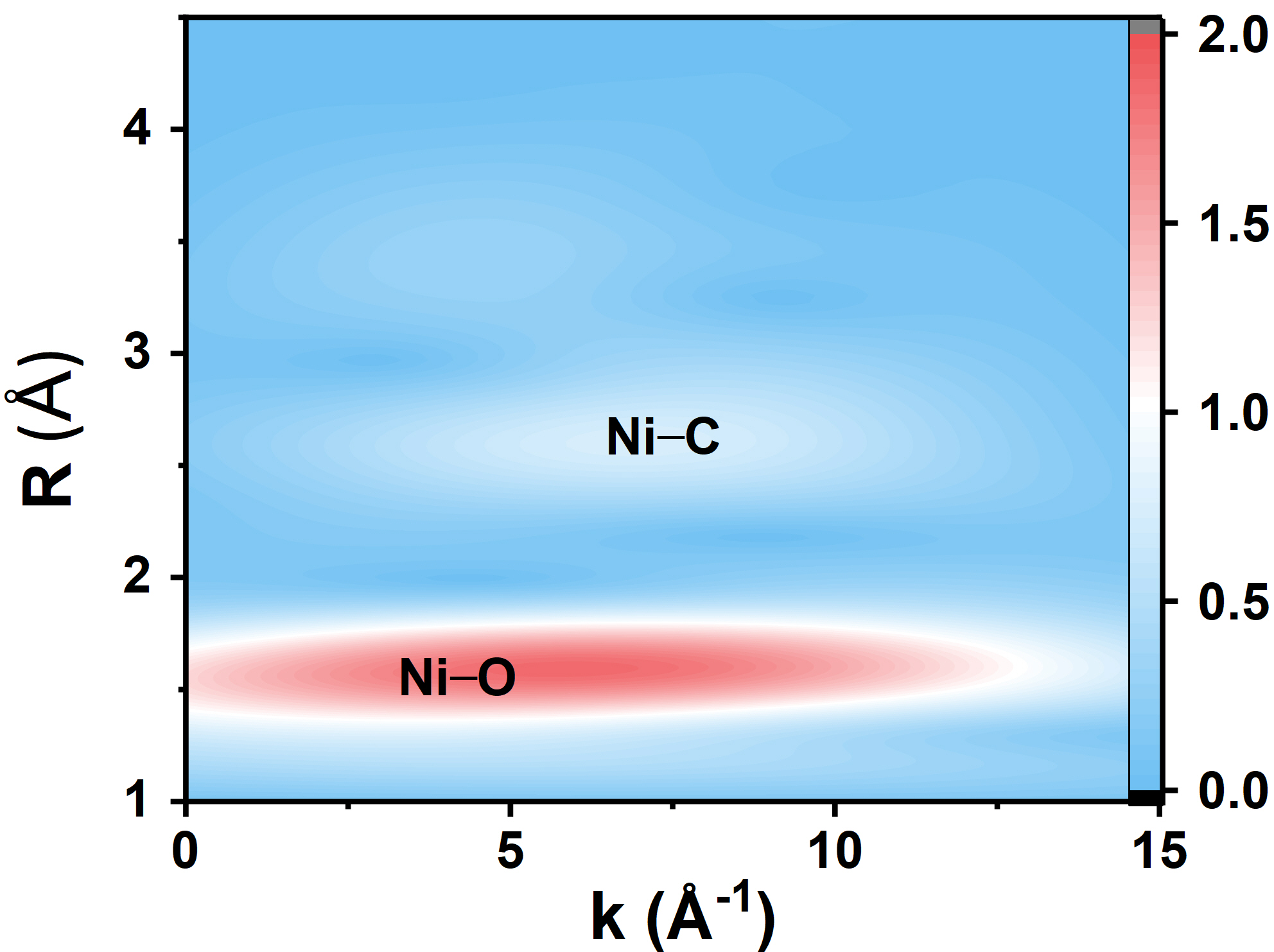


**Figure S10.** WT-EXAFS of Ni-MOF-74 (after).


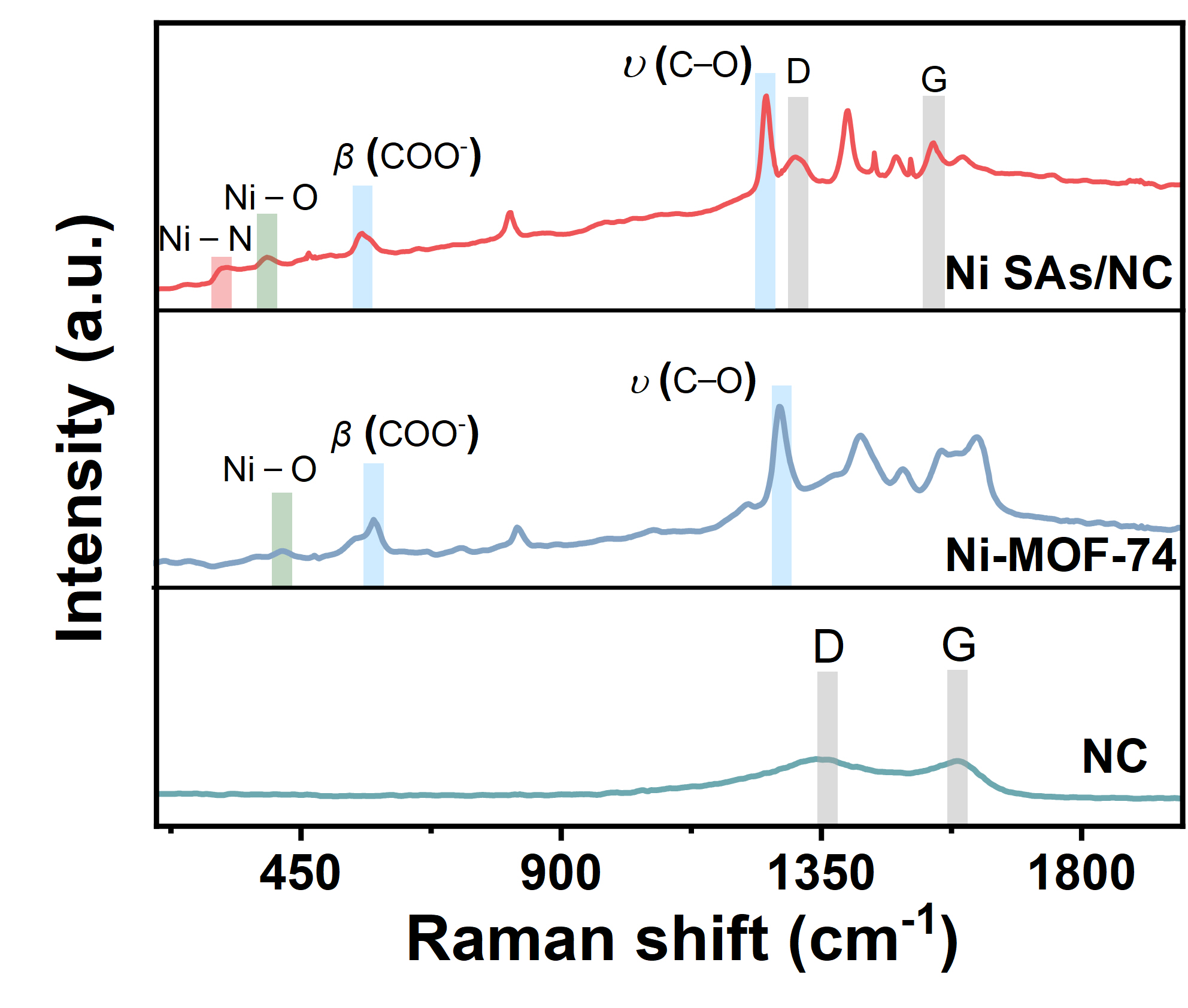


**Figure S11.** Raman spectra of NC, Ni-MOF-74, and Ni SAs/NC.


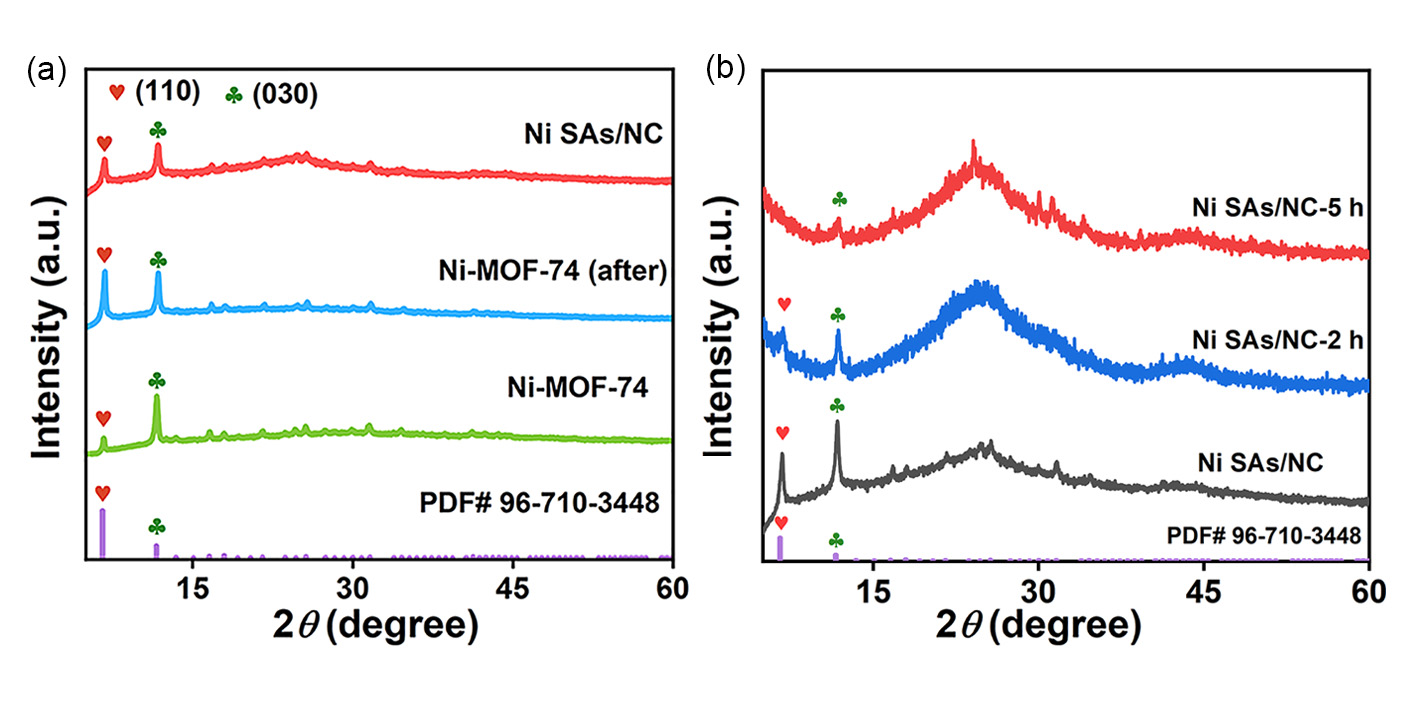


**Figure S12.** (a) XRD patterns of Ni-MOF-74, Ni-MOF-74 after electrolysis (i.e., Ni-MOF-74 (after)), and Ni SAs/NC. (b) XRD patterns of Ni SAs/NC samples obtained at different electrolysis times.


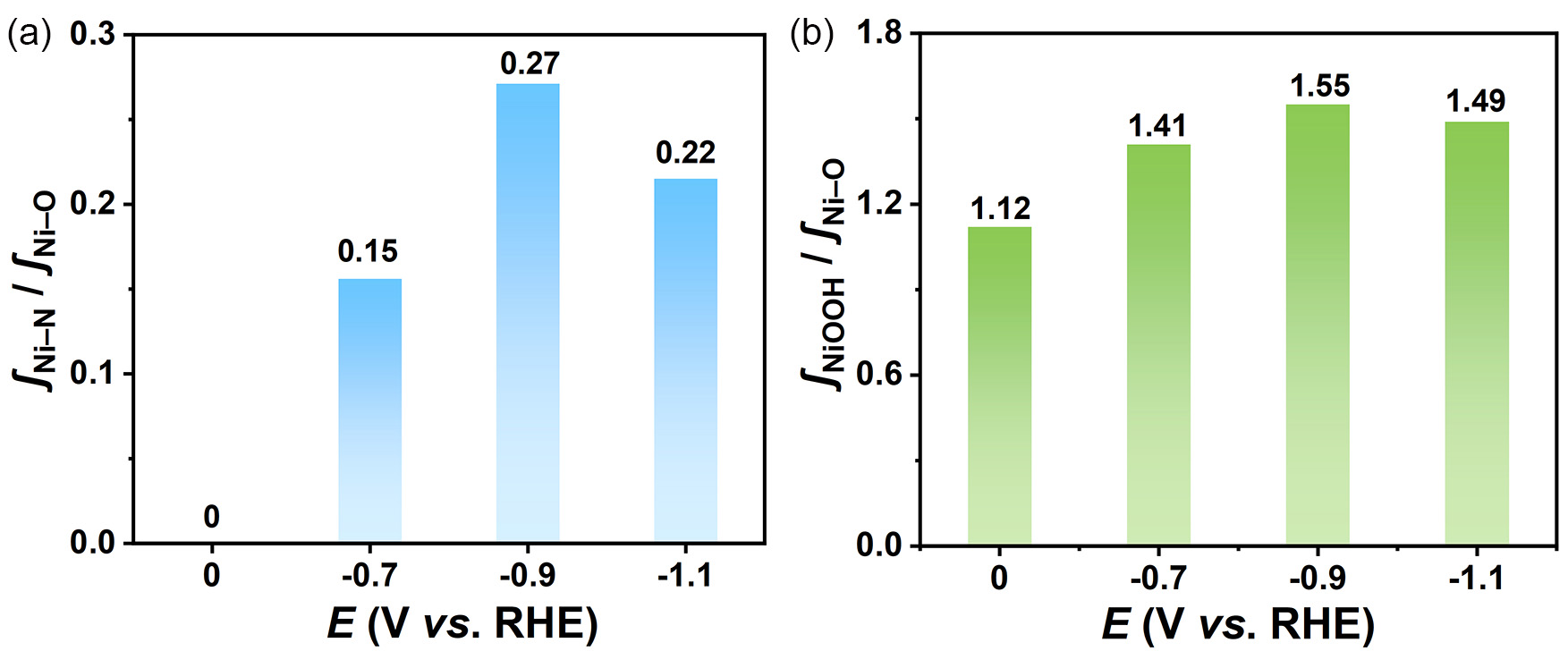


**Figure S13.** (a) The integrated Ni‒N-to-Ni‒O and (b) NiOOH-to-Ni‒O peak area ratio as a function of applied potential for Ni SAs/NC.


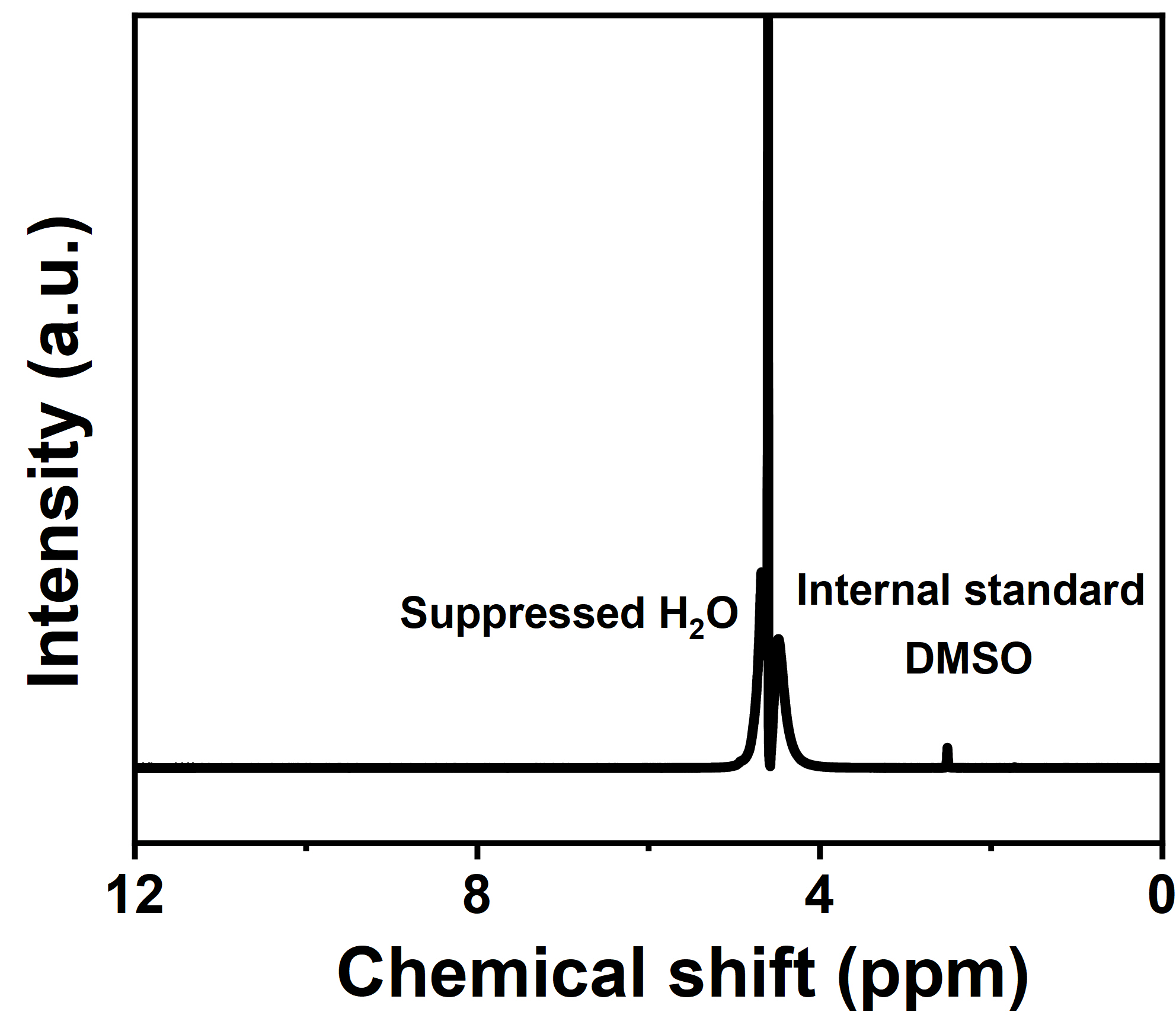


**Figure S14.** ^1^H NMR spectrum of the liquid product after 1 h electrolysis at −0.9 V (*vs*. RHE) using Ni SAs/NC catalyst. Note that DMSO are the internal standard. It can be seen that no liquid product has been detected from the ^1^H NMR spectrum.


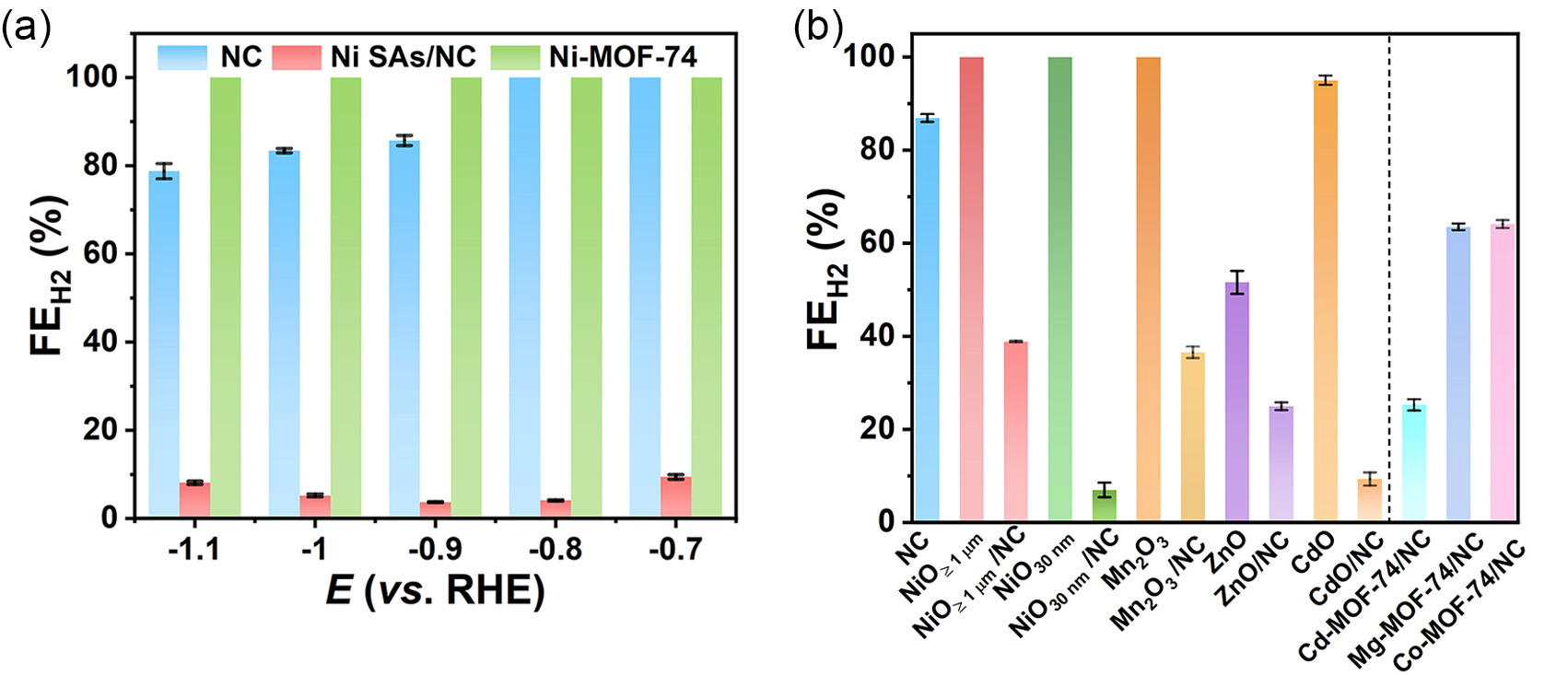


**Figure S15.** (a) FEs toward H_2_ formation (FE_H2_) for Ni SAs/NC, NC, and Ni-MOF-74 at varied applied potentials. (b) FE_H2_ for NC, bare metal oxides, bare MOFs, and SACs resulting from different MOFs and metal oxide precursors.

*
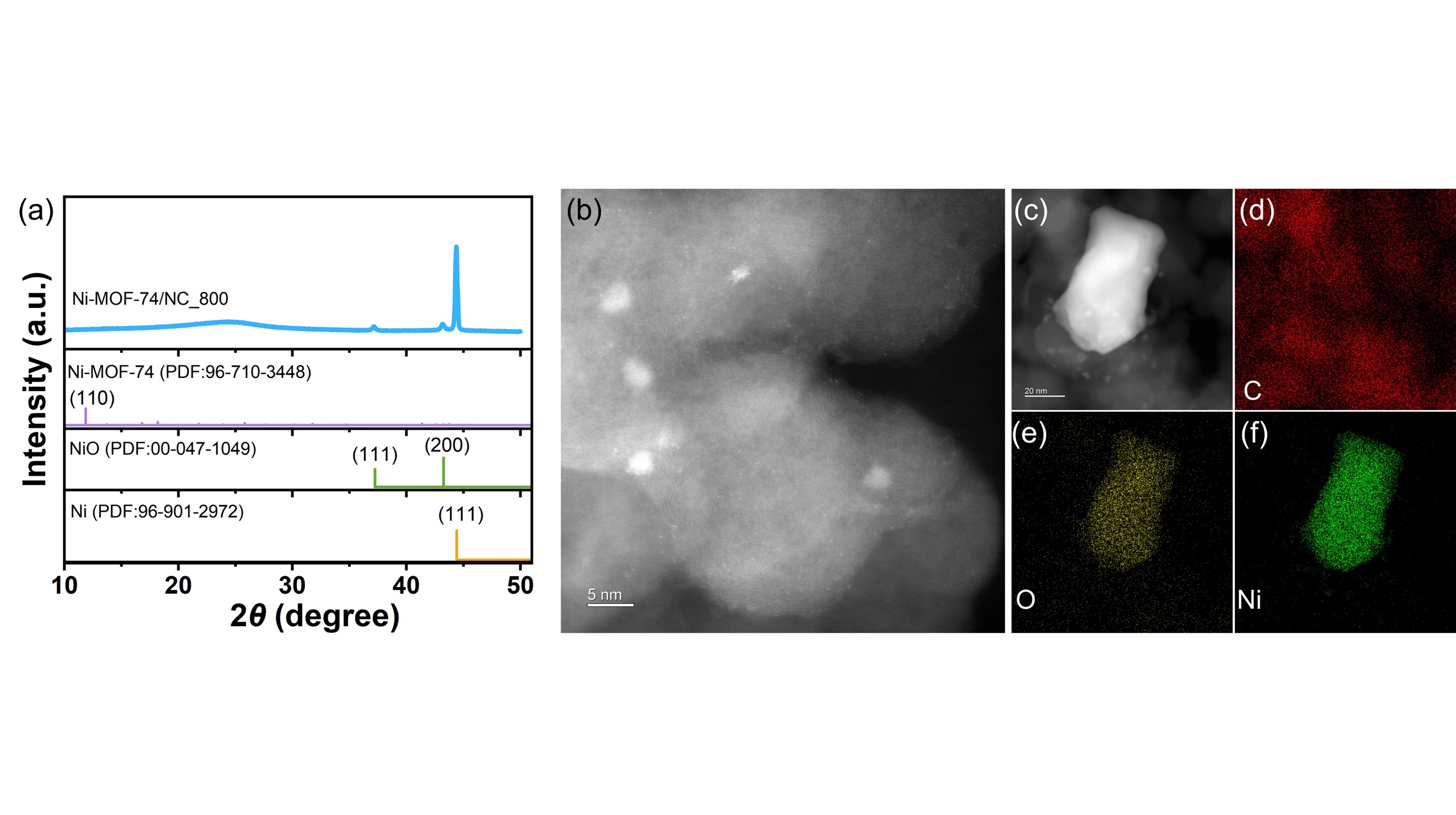
*

**Figure S16.** (a) XRD and (b and c) HAADF-STEM images, and (d‒f) corresponding EDS elemental maps of NiN_x_O_y_/NC obtained by pyrolyzing the mixture of Ni-MOF-74 and NC under Ar at 800 °C for 2 h shown in (c).


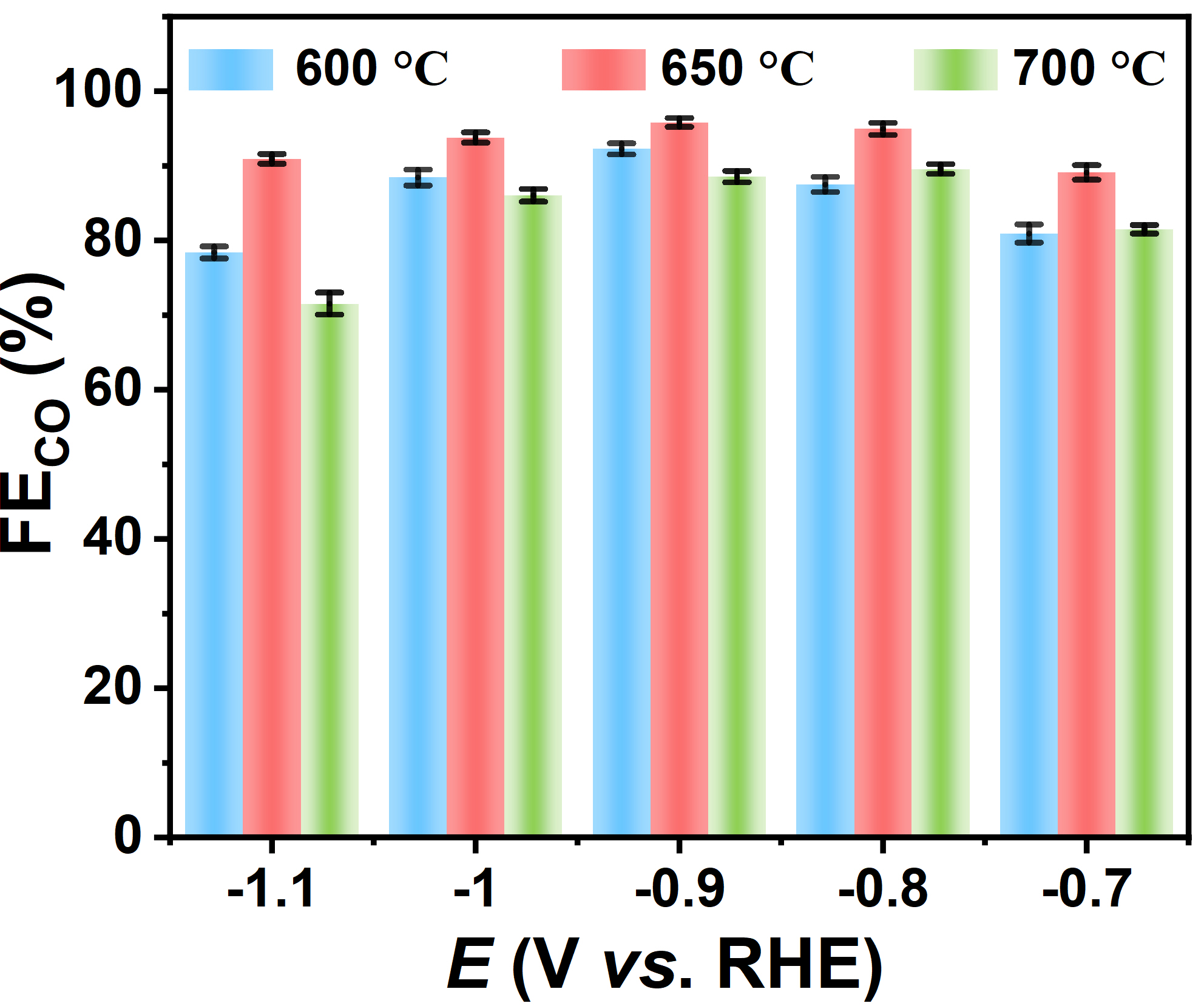


**Figure S17.** FEs toward CO formation for Ni SAs/NC samples synthesized using NC obtained at different pyrolysis temperatures.

*
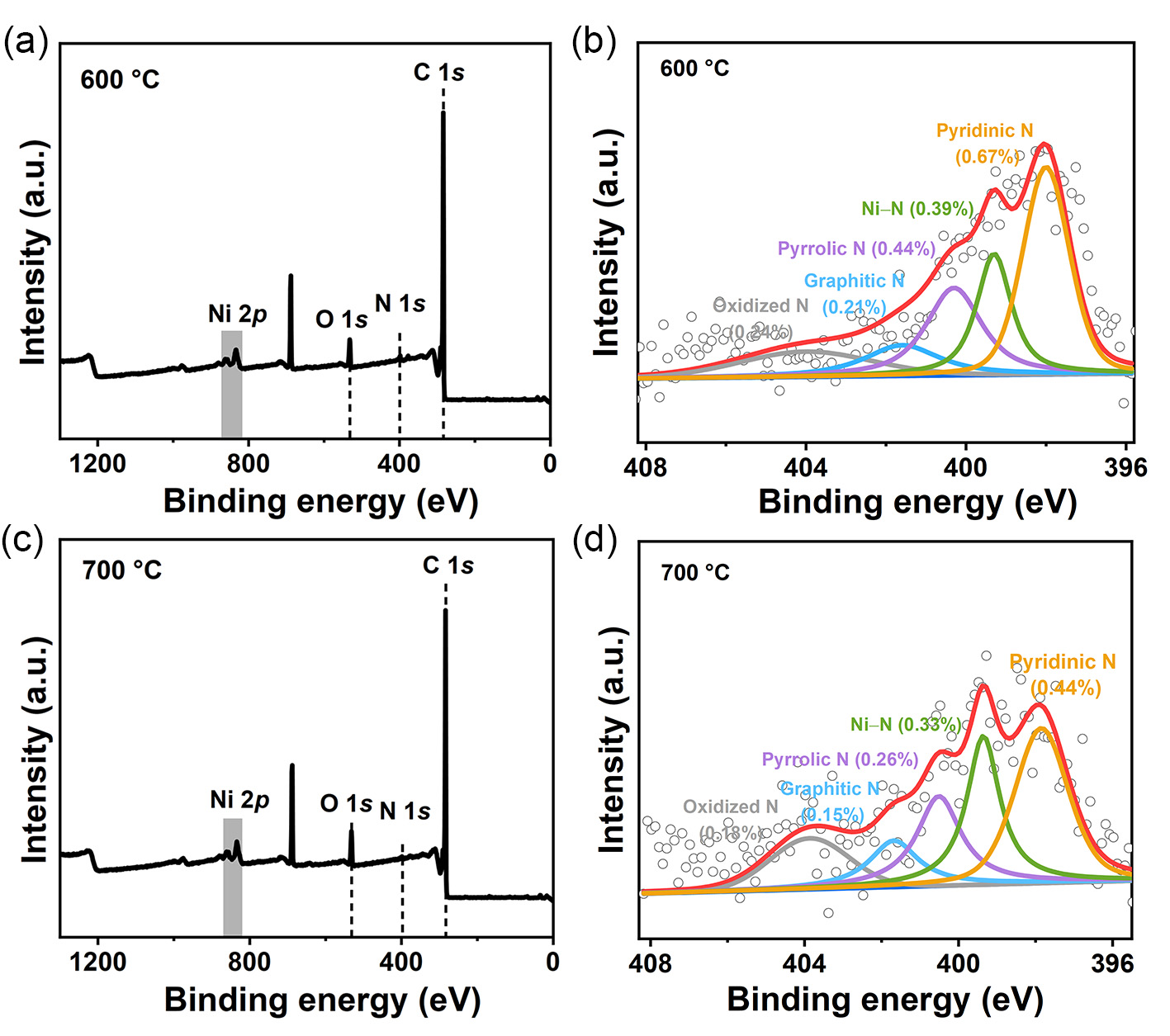
*

**Figure S18.** (a) Wide-survey and (b) N 1*s* XPS spectra for Ni SAs/NC synthesized using NC obtained at 600 ℃. (c) Wide-survey and (d) N 1*s* XPS spectra for Ni SAs/NC synthesized using NC obtained at 700 ℃.


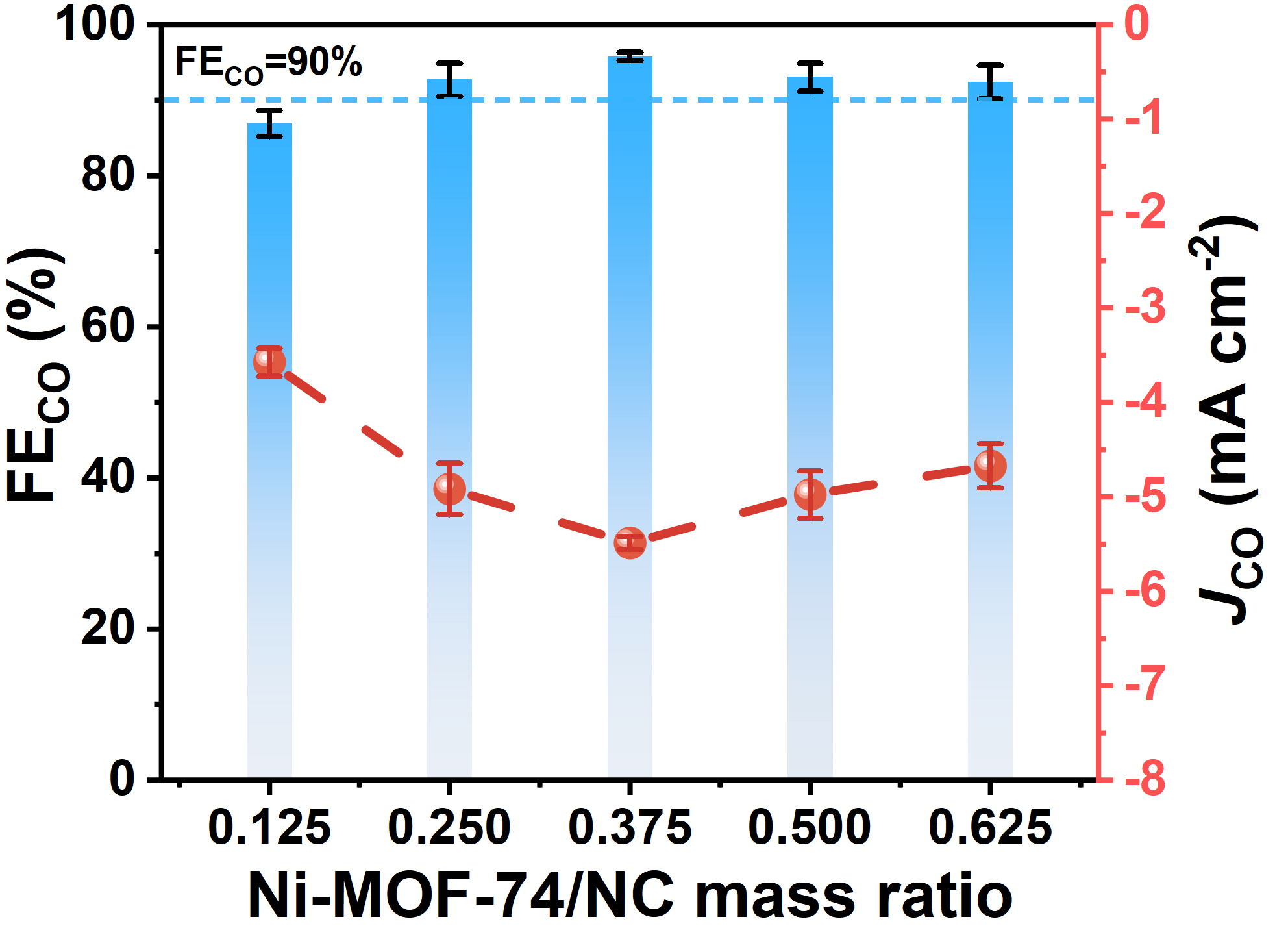


**Figure S19.** FEs and *J* toward CO (*J*_CO_) for Ni SAs/NC synthesized with different mass ratios of Ni-MOF-74 precursor and NC.


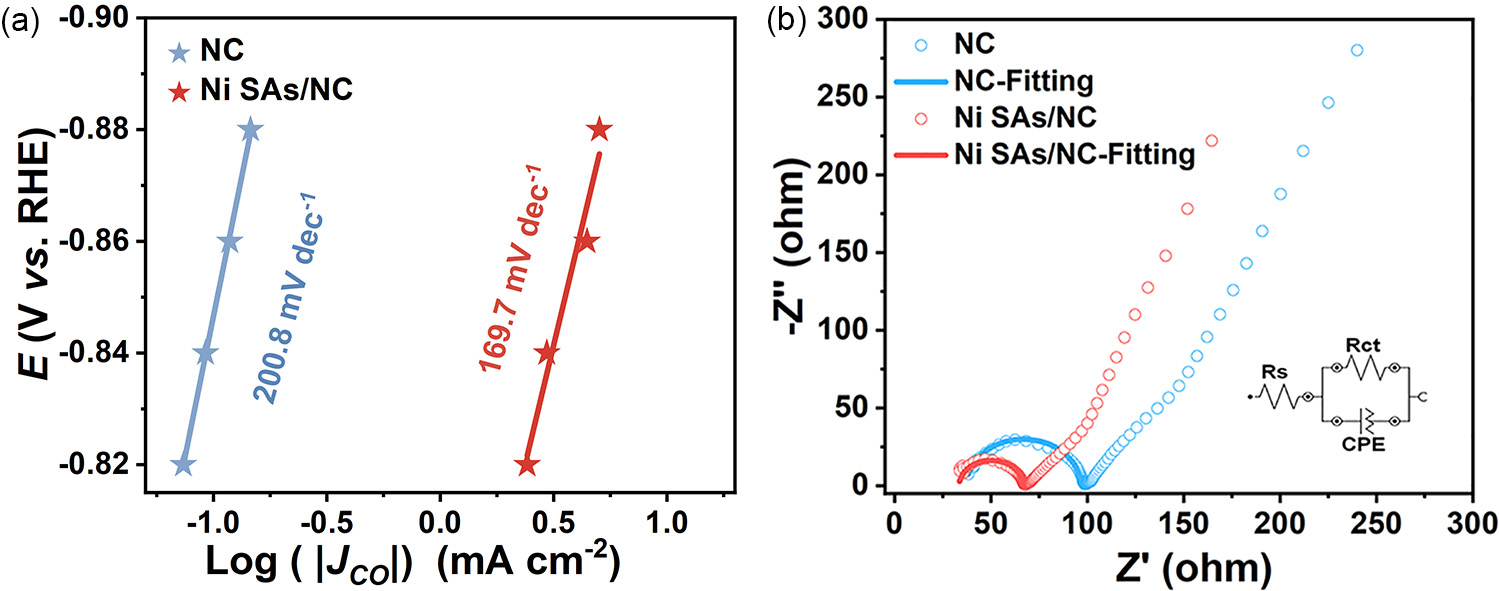


**Figure S20.** (a) Tafel plots and (b) EIS Nyquist profiles of Ni SAs/NC and NC.


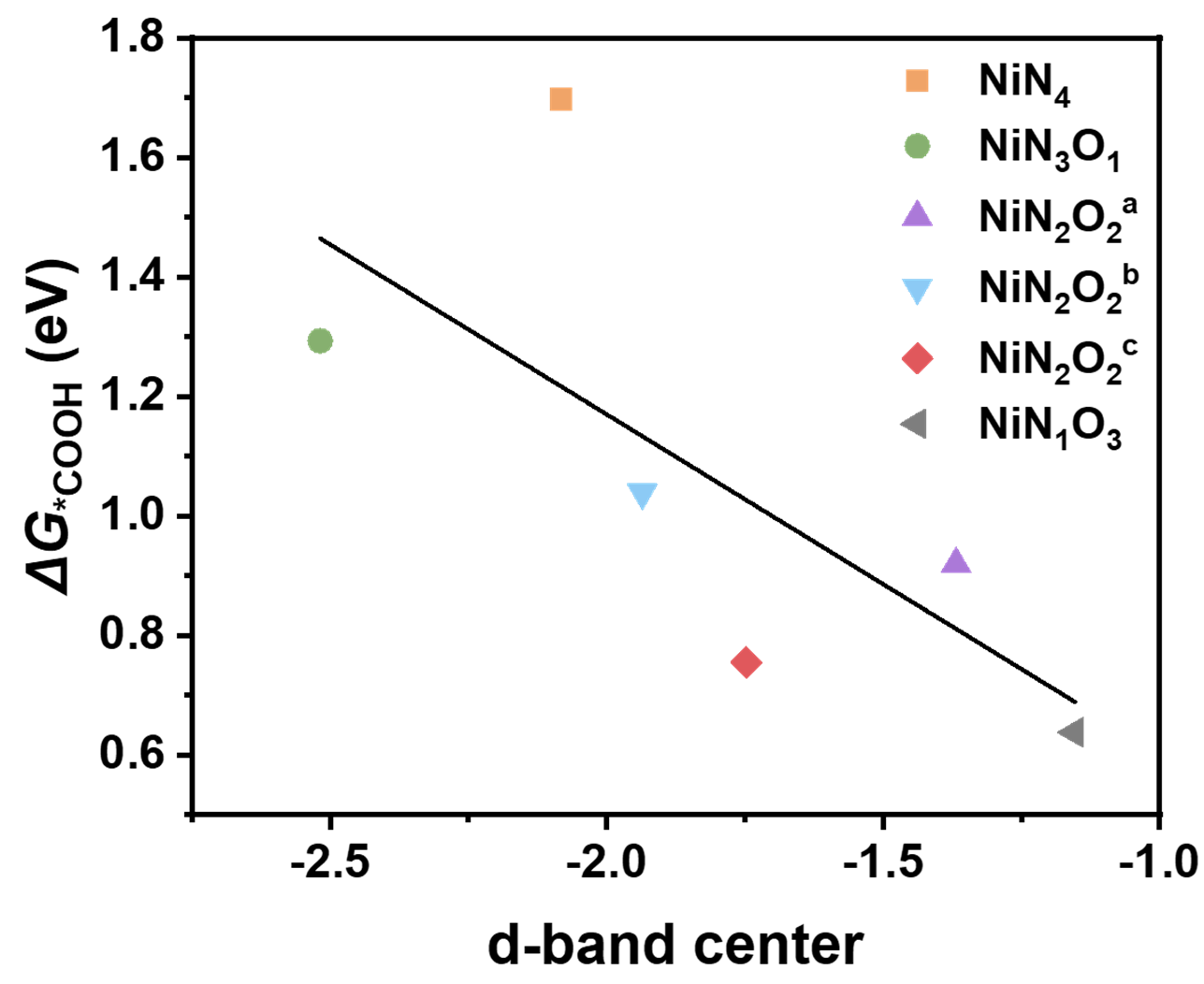


**Figure S21.** The d-band centers and Δ*G*_*COOH_ for six catalysts.

**Table S1.** Structural parameters of EXAFS fitting for Ni foil, Ni-MOF-74 (after), and Ni SAs/NC.

| Sample | Path | CN | *R*, Å | (*σ*^2^, Å^2^) | *ΔE*_0_, eV | *R*-factor, % |
| --- | --- | --- | --- | --- | --- | --- |
| Ni-MOF-74 | Ni−O | 2.3±0.2 | 1.93±0.01 | 0.003±0.001 | –9.9±0.8 | 0.4 |
|  | Ni−O | 3.3±0.2 | 2.08±0.01 | 0.003±0.001 |  |  |
|  | Ni−O | 1.2±0.1 | 2.38±0.01 | 0.013±0.005 |  |  |
|  | Ni−C | 4.0±0.4 | 3.05±0.03 | 0.007±0.003 | 5.7±0.4 |  |
|  | Ni−Ni | 1.8±0.4 | 3.04±0.02 | 0.008±0.002 | 9.4±0.3 |  |
| Ni-MOF-74 (after) | Ni−O | 2.2±0.2 | 1.93±0.01 | 0.003±0.001 | –9.9±0.5 | 1.0 |
|  | Ni−O | 3.1±0.2 | 2.08±0.01 | 0.003±0.001 |  |  |
|  | Ni−O | 1.1±0.1 | 2.36±0.01 | 0.012±0.002 |  |  |
|  | Ni−C | 4.0±0.5 | 3.06±0.03 | 0.003±0.002 | 7.7±0.6 |  |
|  | Ni−Ni | 1.9±0.6 | 3.06±0.03 | 0.009±0.003 | 9.0±0.3 |  |
| Ni SAs/NC | Ni−N/O | 2.3±0.2 | 1.93±0.01 | 0.003±0.001 | –9.3±0.6 | 0.5 |
|  | Ni−N/O | 3.3±0.5 | 2.09±0.01 | 0.003±0.001 |  |  |
|  | Ni−N/O | 1.1±0.1 | 2.37±0.02 | 0.011±0.005 |  |  |
|  | Ni−C | 4.3±0.5 | 3.03±0.01 | 0.003±0.002 | 4.9±0.6 |  |
|  | Ni−Ni | 2.1±0.5 | 3.03±0.02 | 0.012±0.003 | 9.1±0.5 |  |
| Ni foil | Ni−Ni | 12 | 2.48±0.01 | 0.006±0.001 | 6.9±0.4 | 0.2 |

Ni K-edge EXAFS fitting results, in which CN is the average coordination number, *R* is the distance from absorber atom, and *σ*^2^ the Debye–Waller factor. *R*-factor denotes a quality factor of the fitting, and *ΔE*_0_ the energy shift from the absorption edge energy *E*_0_. Fitting parameters: *k* = 3.0 – 9.5; *R* = 1.0 – 3.0; amp = 0.85395.^[11]^

**Table S2.** N species contents for Ni SAs/NC samples synthesized using NC obtained at different pyrolysis temperatures.

| Temperature (℃) | Oxidized N (%) | Graphitic N (%) | Ni−N (%) | Pyrrolic N (%) | Pyridinic N (%) |
| --- | --- | --- | --- | --- | --- |
| 600 | 0.24 | 0.21 | 0.39 | 0.44 | 0.67 |
| 650 | 0.68 | 0.61 | 0.50 | 0.53 | 1.04 |
| 700 | 0.18 | 0.15 | 0.33 | 0.26 | 0.44 |

**Table S3.** Table of calculated Gibbs free energy (∆*G*) of CO_2_ pathways and potential determined steps (PDS) at Ni sites with different coordinated structures.

| Type | PDS | $\Delta\text{G}$ (eV) |
| --- | --- | --- |
| NiN_4_ | * + CO_2_ + H^+^ + *e*^−^ → *COOH | 1.70 |
| NiN_3_O_1_ | * + CO_2_ + H^+^ + *e*^−^ → *COOH | 1.29 |
| NiN_2_O_2_^a^ | * + CO_2_ + H^+^ + *e*^−^ → *COOH | 0.92 |
| NiN_2_O_2_^b^ | * + CO_2_ + H^+^ + *e*^−^ → *COOH | 1.04 |
| NiN_2_O_2_^c^ | * + CO_2_ + H^+^ + *e*^−^ → *COOH | 0.75 |
| NiN_1_O_3_ | *CO → * + CO | 1.32 |

# References

[1] H. Sun, D. Ren, R. Kong, D. Wang, H. Jiang, J. Tan, D. Wu, S. Chen, B. Shen, *Micropor. Mesopor. Mater.* 2019, *284*, 151−160.

[2] Z. Yao, S. Liu, H. Liu, Y. Ruan, S. Hong, T. S. Wu, L. Hao, Y. L. Soo, P. Xiong, M. M. J. Li, A. W. Robertson, Q. Xia, L. X. Ding, Z. Sun, *Adv. Funct. Mater.* 2022, *33*, 2209843.

[3] X. Li, L. Li, Q. Xia, S. Hong, L. Hao, A. W. Robertson, H. Zhang, T. W. B. Lo, Z. Sun, *ACS Sustain. Chem. Eng.* 2022, *10*, 6466−6475.

[4] L. Jiao, W. Yang, G. Wan, R. Zhang, X. Zheng, H. Zhou, S. H. Yu, H. L. Jiang, *Angew. Chem. Int. Ed.* 2020, *59*, 20589−20595.

[5] G. Kresse, J. Furthmüller, *Phys. Rev. B* 1996, *54*, 11169−11186.

[6] J. P. Perdew, K. Burke, M. Ernzerhof, *Phys. Rev. Lett.* 1996, *77*, 3865−3868.

[7] E. Torres, T. P. Kaloni, *Comp. Mater. Sci.* 2020, *171*, 109237.

[8] S. Grimme, J. Antony, S. Ehrlich, H. Krieg, *J. Chem. Phys.* 2010, *132*, 154104.

[9] H. J. Monkhorst, J. D. Pack, *Phys. Rev. B* 1976, *13*, 5188−5192.

[10] V. Wang, N. Xu, J.-C. Liu, G. Tang, W.-T. Geng, *Comput. Phys. Commun.* 2021, *267*, 108033.

[11] Y. Cheng, S. Zhao, B. Johannessen, J. P. Veder, M. Saunders, M. R. Rowles, M. Cheng, C. Liu, M. F. Chisholm, R. De Marco, H. M. Cheng, S. Z. Yang, S. P. Jiang, *Adv. Mater.* 2018, *30*, 1706287.
